# Hallmark Molecular and Pathological Features of POLG Disease are Recapitulated in Cerebral Organoids

**DOI:** 10.1002/advs.202307136

**Published:** 2024-03-06

**Authors:** Anbin Chen, Tsering Yangzom, Yu Hong, Bjørn Christian Lundberg, Gareth John Sullivan, Charalampos Tzoulis, Laurence A. Bindoff, Kristina Xiao Liang

**Affiliations:** ^1^ Department of Clinical Medicine (K1) University of Bergen Bergen 5021 Norway; ^2^ Department of Neurosurgery Xinhua Hospital Affiliated to Shanghai Jiaotong University School of Medicine Shanghai 20092 China; ^3^ Centre for International Health University of Bergen Bergen 5020 Norway; ^4^ Department of Biomedicine University of Bergen Bergen 5009 Norway; ^5^ Department of Pediatric Research Oslo University Hospital Oslo 0424 Norway; ^6^ Neuro‐SysMed Center of Excellence for Clinical Research in Neurological Diseases Haukeland University Hospital Bergen 5021 Norway

**Keywords:** cortical organoids, iPSC, mitochondrial function, neuron, POLG

## Abstract

In this research, a 3D brain organoid model is developed to study POLG‐related encephalopathy, a mitochondrial disease stemming from *POLG* mutations. Induced pluripotent stem cells (iPSCs) derived from patients with these mutations is utilized to generate cortical organoids, which exhibited typical features of the diseases with *POLG* mutations, such as altered morphology, neuronal loss, and mitochondiral DNA (mtDNA) depletion. Significant dysregulation is also identified in pathways crucial for neuronal development and function, alongside upregulated NOTCH and JAK‐STAT signaling pathways. Metformin treatment ameliorated many of these abnormalities, except for the persistent affliction of inhibitory dopamine‐glutamate (DA GLU) neurons. This novel model effectively mirrors both the molecular and pathological attributes of diseases with *POLG* mutations, providing a valuable tool for mechanistic understanding and therapeutic screening for POLG‐related disorders and other conditions characterized by compromised neuronal mtDNA maintenance and complex I deficiency.

## Graphical abstract

This research demonstrates that cerebral organoids derived from iPSCs of *POLG* mutation patients replicate essential aspects of POLG disease, including neuronal loss and mitochondrial dysfunction. Additionally, the study explores the therapeutic potential of metformin, revealing its efficacy in ameliorating organoid phenotypes, suggesting new avenues for treatment strategies in POLG‐related disorders.

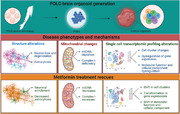

## 1 Introduction

Mitochondrial diseases represent the largest class of inborn errors of metabolism and comprise mainly monogenic disorders that disrupt the process of oxidative phosphorylation (OXPHOS). One of the most common mitochondrial diseases is caused by mutations in the *POLG* gene encoding the catalytic subunit of the mitochondrial DNA polymerase gamma (Pol γ), which is responsible for replication and repair of mitochondrial DNA (mtDNA). Deleterious *POLG* mutations impair mtDNA maintenance and result in quantitative and qualitative mtDNA defects, which in turn cause mitochondrial respiratory chain deficits – mainly affecting complex I and, to a lesser degree, complex IV.^[^ ^1^, ^2^ ^]^ Clinically, diseases with *POLG* mutations manifest a broad phenotypical spectrum ranging from pure myopathies,^[^ ^3^ ^]^ to juvenile syndromes with progressive spinocerebellar ataxia and epilepsy^[^ ^4^, ^5^, ^6^, ^7^ ^]^ and devastating infantile hepatic encephalopathy.^[^ ^8^ ^]^ Moreover, the downstream molecular consequences of *POLG* mutations – defective mtDNA maintenance and complex I deficiency – are also seen in idiopathic forms of age‐related neurodegeneration including Parkinson's disease (PD) and Alzheimer's disease (AD).^[^ ^9^, ^10^, ^11^ ^]^ Disease caused by *POLG* mutations is, therefore, an excellent model for exploring the pathogenic mechanisms by which impaired mtDNA maintenance and complex I deficiency cause neuronal disease, as well as for screening and assessing potential therapies targeting these processes.

The pathophysiological mechanisms underlying neurodegeneration in mitochondrial disease are poorly understood and there are no effective neuroprotective therapies able to delay or arrest disease progression. Advances in this field require the development of model systems that recapitulate the molecular and cellular phenotypes characterizing human disease. Advances in stem cell technology now allow us to generate complex models using patient‐derived induced pluripotent stem cells (iPSCs), reprogrammed into neurons and other brain cell‐types. We previously generated iPSCs from POLG patients and differentiated them into neural stem cells (NSCs) and dopaminergic neurons.^[^ ^12^, ^13^ ^]^ While this traditional 2D iPSC model (monolayer culture) is a powerful and reproducible tool, it does not reflect the complexity and high‐level organization seen at the level of human brain tissue, thus limiting its mechanistic relevance for human disease.^[^ ^14^ ^]^ This limitation can be mitigated by resorting to brain organoids, i.e., 3D structures composed of multiple cell types that can self‐organize to recapitulate embryonic and tissue development in vitro. Brain organoids have been shown to outperform 2D cell culture methods in reflecting the functional, structural, and geometric characteristics of brain tissue.^[^ ^15^, ^16^, ^17^ ^]^ Moreover, since brain organoids can be generated in large numbers, they also have advantages over animal models in drug screening studies. Given that drugs ameliorating neurological disorders can be effective in animal models but fail in clinical trials,^[^ ^18^, ^19^ ^]^ this emphasizes the need for human cell‐based systems to evaluate drug efficacy.

Metformin, a drug primarily used for the management of type 2 diabetes, has demonstrated potential therapeutic benefits in a variety of other conditions including cancer, cardiovascular diseases, and neurodegenerative disorders.^[^ ^20^ ^]^ Its proposed neuroprotective effects can be attributed to several mechanisms: primarily, metformin activates AMP‐activated protein kinase (AMPK), a key regulator of cellular energy homeostasis, which in turn can enhance mitochondrial function and cell survival – critical factors in conditions such as POLG‐related disorders characterized by mitochondrial dysfunction.^[^ ^21^ ^]^ In addition, metformin possesses anti‐inflammatory properties, exerted through the inhibition of the NF‐κB signaling pathway,^[^ ^22^ ^]^ which might help in managing the inflammatory aspects of neurodegenerative diseases. Another potential benefit of metformin in this context is its ability to stimulate autophagy, a cellular recycling process that maintains neuronal health by degrading damaged proteins and organelles.^[^ ^23^ ^]^ This effect could potentially contribute to the clearance of disease‐associated pathogenic proteins and improve neuronal function. Nonetheless, additional research and clinical trials are necessary to validate the effectiveness and safety of metformin in treating neurodegenerative disorders, particularly those resulting from mitochondrial dysfunction such as POLG‐related diseases.

We present a novel iPSC‐derived cortical organoid model using cells derived from two patients with POLG encephalopathy, one harboring the homozygous c2243 G > C (resulting in amino acid change p. W748S) and one carrying compound heterozygous c.1399 G > A and c2243 G > C mutations (resulting in amino acid changes p. A467T and p. W748S). Our patient‐derived organoids exhibited striking morphological abnormalities, respiratory complex I deficiency, and mtDNA depletion, accurately recapitulating the key histological and molecular features observed in diseases with *POLG* mutations in the human brain. Notably, we found that supplementation with metformin effectively mitigated these phenotypes, offering a potential therapeutic avenue for POLG‐related disorders.

## 2 Results

### 2.1 Generation of Cortical Organoids from iPSCs

We generated iPSCs from the skin fibroblasts of two patients with *POLG* mutations: one patient carrying homozygous c.2243 G > C, p.W748S (WS5A) and another patient with compound heterozygous c.1399 G > A, p.A467T and c.2243 G > C, p.W748S (CP2A).^[^ ^12^, ^13^, ^24^ ^]^ We also included iPSCs generated from skin fibroblasts from two neurologically healthy individuals as controls, as previously described in our reports.^[^ ^12^, ^13^ ^]^ The patient's symptoms included progressive spinocerebellar ataxia, peripheral neuropathy, migraine‐like headaches, and extraocular myopathy, leading to progressive external ophthalmoplegia, as reported in the previous study.^[^ ^7^ ^]^ The reprogrammed patient iPSCs exhibited standard karyotyping compared to the original fibroblasts, alongside positive expression of SOX2, OCT4, and NANOG (Figures S2 and S3, Supporting Information).

**Figure 1:**
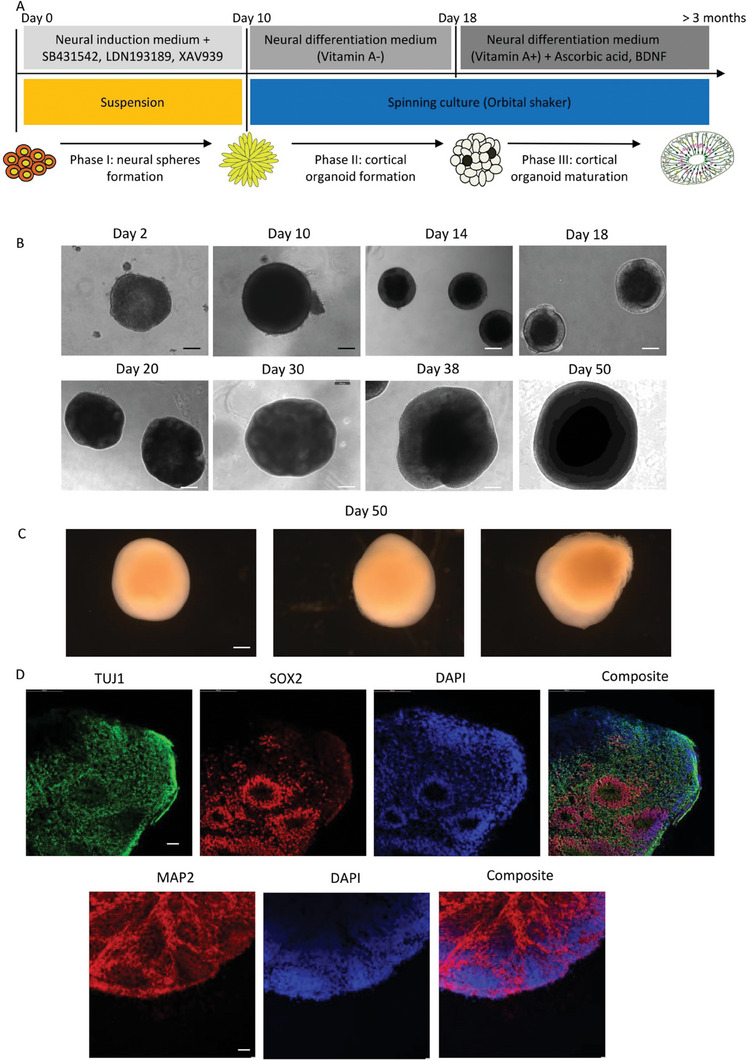
Generation of cortical organoids from iPSCs. A) The differentiation protocol consisted of three phases. In Phase I, neural induction and neural sphere formation were achieved by generating embryoid bodies in stationary suspension 3D culture using the dual SMAD inhibition and canonical Wnt inhibition approach. In Phase II, cortical organoid formation was initiated by transferring the cells into spinning culture using an orbital shaker and culturing them in neural differentiation medium without vitamin A to promote regionalization factors and cortical organization. In Phase III, cortical organoid maturation was facilitated by maintaining the organoids in neural differentiation medium supplemented with vitamin A, BDNF, and ascorbic acid for long‐term neural maturation. B) Representative phase contrast images were captured at various time points during the differentiation process, including day 2, 10, 14, 18, 20, 30, 38, and 50. These images displayed the changing cell morphology throughout the differentiation process. The black scale bar represents 100 µm, and the white scale bar represents 300 µm. C) The morphology of three individual organoids on day 50 of differentiation was examined. The image displayed the diversity in size and structure among the organoids. The scale bar represents 400 µm. D) Representative immunofluorescent imaging was performed on cryo‐sectioned organoids on day 30 of differentiation from the control CCD‐1079Sk iPSCs. The staining revealed the presence of the newborn neural marker TUJ1 (green), neural progenitor marker SOX2 (red), and mature neural marker MAP2 (red). Nuclei were stained with DAPI (blue). The scale bar represents 100 µm.

Cortical organoids were generated by inducing neural differentiation of iPSCs through the formation of embryoid bodies (EBs) in suspension culture. This was achieved using a neural induction protocol that involved dual SMAD inhibition and canonical Wnt inhibition (**Figure** **1**A).^[^ ^12^, ^25^ ^]^ This led to the creation of cortical region brain tissues, termed as cortical organoids; this took 2 days for EB formation, 10 days for neural identity appearance and 20–30 days for definitive brain region formation (Figure 1B). On day 50, the organoids matured into large, complex heterogeneous tissues up to 3 mm in diameter (Figure 1C) that survived for 4–5 months when kept in a spinning rotator.

**Figure 2:**
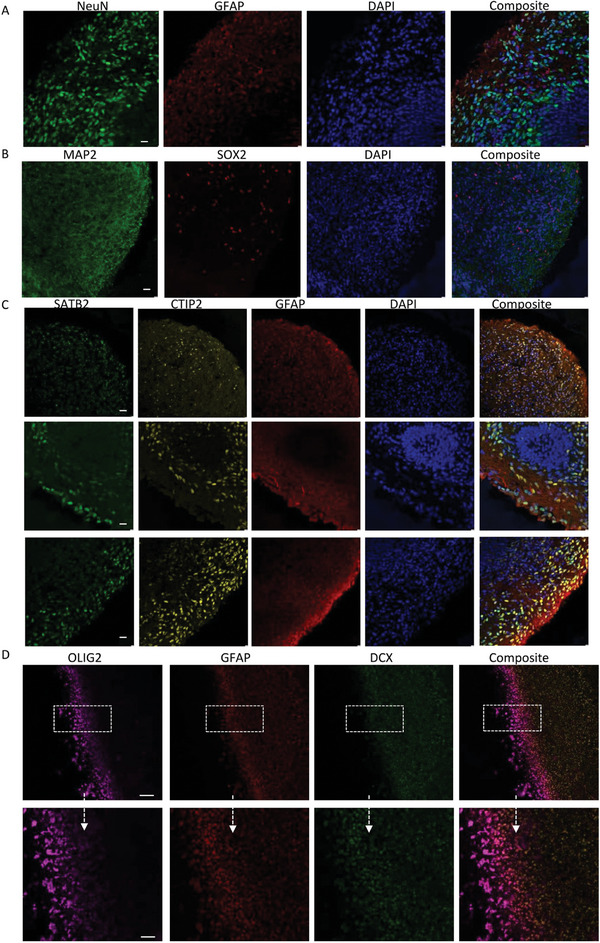
Characterization of cortical organoids from iPSCs on day 90. A) Representative immunofluorescent imaging of cryo‐sectioned organoids cortical organoids on day 90 derived from the control CCD‐1079Sk iPSCs revealing the presence of the astrocyte marker GFAP (red) and the mature neural marker NeuN (green). Nuclei were counterstained with DAPI (blue). The scale bar represents 100 µm. B) Representative immunofluorescent imaging of cryo‐sectioned cortical organoids derived from the control CCD‐1079Sk iPSCs on day 90 showed the expression of the mature neural marker MAP2 (green) and the neural progenitor marker SOX2 (red). Nuclei were counterstained with DAPI (blue). The scale bar represents 100 µm. C) Cryo‐sectioned cortical organoids derived from the control CCD‐1079Sk iPSCs at day 90 displayed cortical pyramidal neuronal markers SATB2 (green) and CTIP1 (yellow), along with the astrocyte marker GFAP (red). Nuclei were counterstained with DAPI (blue). The scale bar represents 100 µm. D) Representative immunofluorescent imaging of cryo‐sectioned cortical organoids derived from the control CCD‐1079Sk iPSCs at day 90 revealed stratified layers containing the oligodendrocyte marker OLIG2 (purple), the astrocyte marker GFAP (red), and the neural marker DCX (green). Nuclei were counterstained with DAPI (blue). The scale bar represents 100 µm.

At different stages of development, we noted different organoid characteristics. At the early stage (30 days), small organoids expressed the newborn neural marker Tubulin beta III (TUJ1), ventricular zone (VZ) marker SOX2, as well as the mature neural marker MAP2 (Figures 1D and **2**A). By 60 days (intermediate stage), organoids showed more pronounced SOX2 expression (Figure S4B, Supporting Information). By the late stage (70 days), large neural tubes expressing SOX2 and MAP2 were observed in the outer layers (Figure S4C, Supporting Information). An increase in SOX2 levels was noted from day 30 to day 60 and day 70, while MAP2 levels remained stable (Figure S4D,E, Supporting Information).

**Figure 3:**
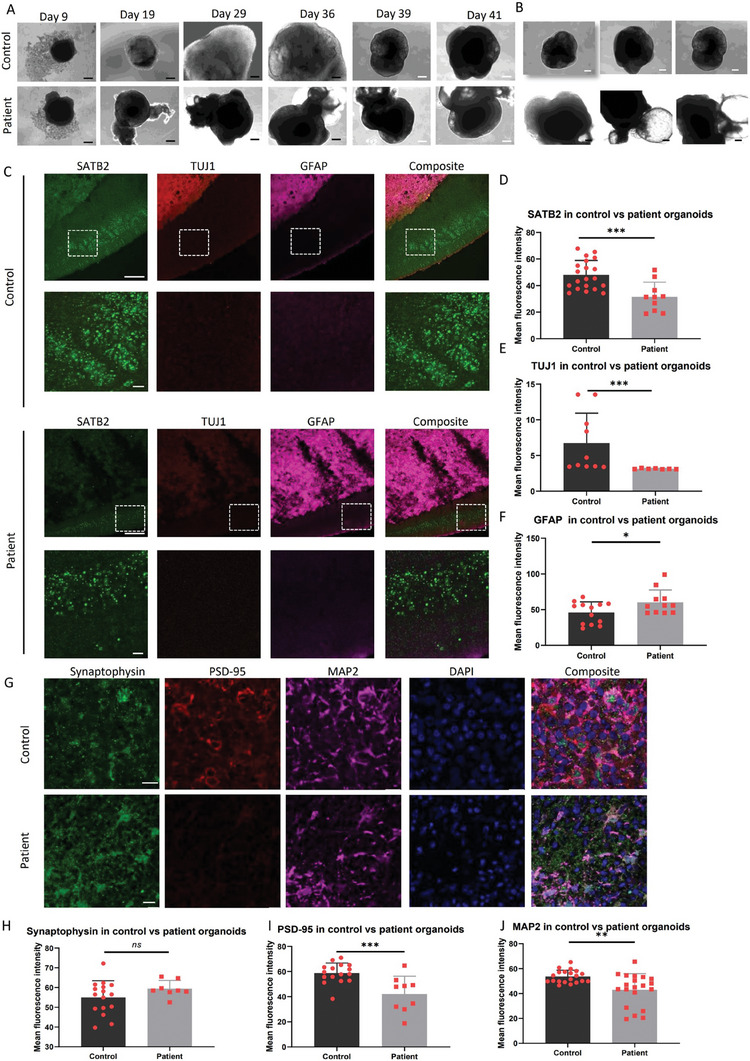
Comparison of lineage markers cortical organoids from control and patient iPSCs. A) Cell morphology was assessed using phase contrast imaging at day 9, 19, 29, 36, 39, and 41 of differentiation in both control Detroit 551 and patient CP2A cortical organoids. The black scale bar represents 100 µm, and the white scale bar represents 100 µm. B) Morphology of three individual organoids on day 36 of differentiation was examined in both control Detroit 551 and patient CP2A cortical organoids. The scale bar represents 300 µm. C) Cryo‐sectioned cortical organoids Representative immunofluorescent imaging of staining for the cortical pyramidal neuronal marker SATB2 (green), newborn neural marker TUJ1 (green), and reactive astrocyte marker GFAP (red) for cryo‐sectioned organoids at 90 days in both control Detroit 551 and patient CP2A cortical organoids. The scale bar represents 100 µm. D–F) Quantitative measurements were performed to assess the expression levels of D) SATB2, E) TUJ1, and F) GFAP on day 90 in control and patient cortical organoids. The y‐axis represents the mean fluorescence intensity. The data were presented as mean ± SD. Significance levels are indicated for P values less than 0.05, with * indicating *P* < 0.05, *** indicating *P* < 0.001. The information on sample size (n), probability (P) value, and the specific statistical test for each experiment was presented in Tables S5 and S9 (Supporting Information). G) Representative immunofluorescent imaging of cryo‐sectioned cortical organoids stained for the presynaptic marker Synaptophysin (green), postsynaptic marker PSD‐95 (red), and mature neural marker MAP2 (purple) from both control Detroit 551 and patient CP2A cortical organoids. Nuclei were counterstained with DAPI (blue). The scale bar represents 50 µm. H–J) Quantitative measurements were conducted to evaluate the expression levels of Synaptophysin H), PSD‐95 I), and MAP2 J) in control and patient cortical organoids. The y‐axis represents the mean fluorescence intensity. The data were presented as mean ± standard deviation (SD)D. Significance levels are indicated for P values less than 0.05, with ** indicating *P* < 0.01, *** indicating *P* < 0.001. Results not reaching statistical significance are denoted as ns (not significant). The information on sample size (n), probability (P) value, and the specific statistical test for each experiment was presented in Tables S5 and S9 (Supporting Information).

Finally, at day 90, the organoids largely consisted of neurons positive for the mature neural marker NeuN, located in the outer layers (Figure 2A). Small proportions of cells expressed GFAP (**Figure** **3**A), MAP2, and SOX2 (Figure 2B). Cortical pyramidal neuronal markers SATB2 and CTIP2 were found in the middle and upper layers of the organoids (Figure 2C), alongside stratified expressions of oligodendrocyte marker oligodendrocyte transcription factor 2 (OLIG2), astrocyte marker GFAP, and neural marker doublecortin (DCX) (Figure 2D).

We concluded therefore, that this process effectively generated 3D cortical organoids comprising neural tubes, cortical neurons, glial astrocytes, and oligodendrocytes.

### 2.2 POLG‐Patient Cortical Organoids Demonstrated Significant Structural Alterations, Marked by Neuronal Loss and an Increase in Astrocytosis

In contrast with control samples that produced orderly neuroepithelial tissues and subsequently matured into regular cortical organoids, POLG patient‐derived samples showed a marked irregularity in their neuroepithelial tissue. These samples matured into structures resembling brain tissue, but without the complete organization seen in control cortical organoids. Aberrant growth was observed throughout the EB development (Figure 3A).

Specific abnormalities became evident at different developmental stages. At 36 days, peripheral neuroepithelial regions of patient‐derived tissue exhibited prominent, fluid‐filled cavity (Figure 3B). By day 90, patient cortical organoids demonstrated a significant reduction in ventricle‐like structures and neuroepithelial layer thickness compared to control cortical organoids (Figure 3C). Furthermore, staining for cortical neuron marker SATB2 was considerably diminished in patient‐derived cortical organoids (Figure 3C,D), as was the case for neuronal marker TUJ1 (Figure 3C,E). In contrast, staining for activated astrocyte marker GFAP was noticeably elevated in patient cortical organoids (Figure 3C,F).

Further analysis was conducted with the presynaptic marker synaptophysin, the postsynaptic marker PSD‐95 and mature neural marker MAP2. When compared to controls, patient cortical organoids showed a marked reduction in the levels of PSD‐95 (Figure 3G,I) and MAP2 (Figure 3G,J), while synaptophysin levels remained comparable with control samples (Figure 3G,H).

In summary, the POLG patient‐derived cortical organoids were distinguished by morphological differences, a reduction in neuronal markers and the excitatory postsynaptic marker, along with an increased presence of astrocytes.

### 2.3 Patient Cortical Organoids Display Altered Expressions of Mitochondrial Proteins

Our prior research highlighted that those patients carrying the same mutations as those modelled in the organoids showed neuronal mtDNA depletion starting in early infancy. This condition is further characterized by progressively increasing levels of mtDNA major arc depletions and respiratory complex I deficiency.^[^ ^24^ ^]^ This observation was replicated in iPSC‐derived 2D NSCs,^[^ ^12^ ^]^ dopaminergic neurons^[^ ^13^ ^]^ and astrocytes^[^ ^26^ ^]^ where we noted both complex I deficiency and mtDNA depletion.

**Figure 4:**
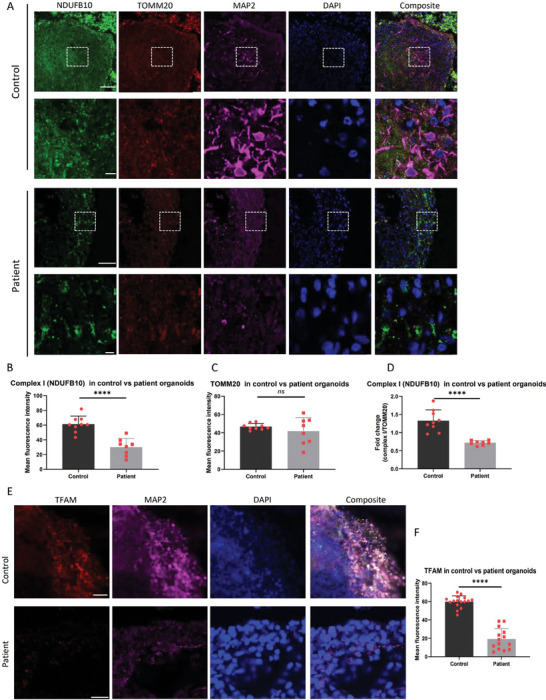
Comparison of mitochondrial related proteins in cortical organoids from control and patient iPSCs. A) Representative immunofluorescent imaging of staining for the complex I subunit NDUFB10 (green), outer mitochondrial membrane TOMM20 (red), and mature neural marker MAP2 (purple) for the cryo‐sectioned cortical organoids at 90 days of differentiation from both control Detroit 551 and patient CP2A iPSCs. The scale bar represents 100 µm. B–D) Quantitative measurements were performed to assess the expression levels of NDUFB10, TOMM20, and MAP2 in control and patient cortical organoids. The y‐axis represents the mean fluorescence intensity. The data were presented as mean ± SD. Significance levels are indicated for P values less than 0.05, with **** indicating *P* < 0.000. Results not reaching statistical significance are denoted as ns (not significant). The information on sample size (n), probability (P) value, and the specific statistical test for each experiment was presented in Tables S5 and S9 (Supporting Information). E) Representative immunofluorescent imaging of staining for the mitochondrial transcription factor A (TFAM), neural progenitor marker SOX2, and mature neural marker MAP2 for cryo‐sectioned organoids at 90 days of differentiation from both control Detroit 551 and patient CP2A cortical organoids. Nuclei were counterstained with DAPI (blue). The scale bar represents 100 µm. F) Quantitative measurements were conducted to evaluate the expression level of TFAM in control and patient cortical organoids. The y‐axis represents the mean fluorescence intensity. The data were presented as mean ± SD. Significance levels are indicated for P values less than 0.05, with **** indicating *P* < 0.000. Results not reaching statistical significance are denoted as ns (not significant). The information on sample size (n), probability (P) value, and the specific statistical test for each experiment was presented in Tables S5 and S9 (Supporting Information).

Here, we explored whether these alterations were also manifested in cortical organoids. Complex I levels were examined by conducting multiple immunostainings for the complex I subunit NDUFB10, the mitochondrial mass marker TOMM20, and the mature neuron marker MAP2. We observed a significant decrease in complex I levels in neuronal component of patient cortical organoids compared to controls (**Figure** **4**A,B). However, TOMM20 levels showed no significant change (Figure 4A,C) suggesting that this was not due simply to loss of mitochondrial mass. The NDUFB10/TOMM20 ratio, an indicator of complex I per mitochondrion, was substantially lower in patient samples, signaling a loss of complex I (Figure 4D). Moreover, when we indirectly assessed mtDNA levels using immunostaining for mitochondrial transcription factor A (TFAM), patient cortical organoids showed notably lower TFAM levels compared to controls, suggesting that there was indeed mtDNA depletion (Figure 4E,F).

In conclusion, these findings underscore that iPSC‐derived cortical organoids from POLG patients faithfully replicate the key molecular features seen in patient neurons, encompassing neuronal loss, complex I deficiency, and mtDNA depletion.

### 2.4 Single‐Cell Transcriptomics Reveal Multiple Neuronal Cell Types in Cortical Organoids

We employed single cell RNA sequencing (scRNA‐seq) to examine the cellular composition and gene expression profiles of the cortical organoids from control iPSCs. We used 4–6 organoids per group for analysis. Our study utilized age and gender‐matched control cortical organoids derived from Detroit 551 iPSCs as a comparison group. After applying strict filtering criteria, we analyzed a total of 5369 cells that expressed 24576 genes in the control organoid (Table S6, Supporting Information).

We examined the various cell populations based on cluster gene markers and the expression of known marker genes. The cortical organoids of cell type enrichment analysis in scRNA‐seq analysis could be subcategorized further into dopamine‐glutamate (DA GLU) neuron (a distinctive subset of dopaminergic neurons in the ventral tegmental area that also release glutamate)^[^ ^27^ ^]^‐related genes (*SCG2*, *NSG2*, *MMP3*, *ATP1B1*, *RTN4*, *NEGR1*, *HSP90AA1*, *CELF4*, *LMO3*), dopaminergic neurons (*NEUROD6*, *TMSB10*, *TUBA1A*, *SOX4*, *STMN2*, *PTMA*, *NREP*, *SOX11*, *MLLT11*), ependymal cells (*CA4*, *WLS*, *MGST1*, *PRTG*, *PLS3*, *RSPO2*, *FAM122B*, *WNT2B*, *CDO1*), GABAergic neurons (*RAB8A*, *PIGK*, *ZDHHC5*, *FZD3*, *CCM2*, *MRPL47*, *PNRC1*, *RHBDD2*, *CDC42SE1*), glutaminergic neurons (*MT‐CYB*, *MT‐ND5*, *NEFM*, *MT‐ATP6*, *MT‐ND4*, *NEFL*, *MTND3*, *MT‐ND2*, *MT‐CO3*), and neural progenitor cells (*RPS27*, *FTL*, *RPL21*, *FTH1*, *RPL15*, *RPL13A*, *RPS5*, *CDKN1A*, *VIM*) were also detected, along with radial glial cells (*PTN*, *C1orf61*, *VIM*, *CLU*, *HES1*, *HSPB1*, *MDK*, *DBI*, *LINC01158*) (Table S7, Supporting Information).

**Figure 5:**
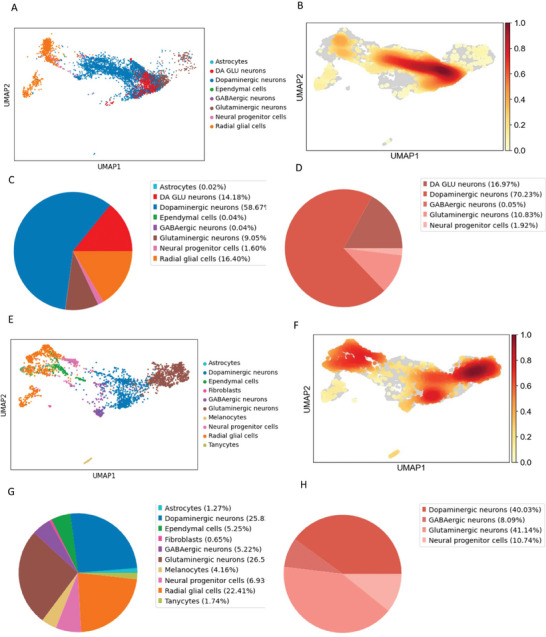
Comparison of single‐cell transcriptomic profiling in cortical organoids from control and patient iPSCs. A,B) Cell clusters in 3‐month‐old organoids derived from control iPSCs were visualized using the scMRMA and UMAP algorithm A), and their density is depicted in B). C,D) The percentage distribution of all cell clusters C) and the neuro population D) in organoids at 3‐months old, which were derived from control iPSCs, were represented with the PCA algorithm. E,F) Cell clusters in 3‐month‐old organoids derived from patient iPSCs were visualized using the scMRMA and UMAP algorithm E), and their density is shown in F). G,H) The percentage distribution of all cell clusters G) and the neuro population H) in organoids at 3‐months old, which were derived from patient iPSCs, were represented with the PCA algorithm.

By applying Uniform Manifold Approximation and Projection (UMAP) analysis, we identified eight distinct cell population clusters (**Figure** **5**A–D). The control cortical organoid consisted of 0.02% astrocytes, 14.18% DA GLU neurons, 58.67% dopaminergic neurons, 0.04% ependymal cells, 0.04% GABAergic neurons, 9.05% glutaminergic neurons, 1.60% neural progenitor cells, and 16.40% radial glial cells (Figure 5C and Figure S5, Supporting Information). Further classification of the neuronal populations revealed that 16.97% were DA GLU neurons, 70.23% were dopaminergic neurons, 0.05% were GABAergic neurons, 10.83% were glutaminergic neurons, and 1.92% were neural progenitor cells (Figure 5D).

Pathway enrichment analyses on the neuronal population (DA GLU neurons and other neurons) affirmed the enrichment of gene sets linked to neural development processes, including axon development, neural migration, generation of neurons, regulation of neural differentiation, neural tube development, axonogenesis, and synaptic transmission (Figures S6 and S7, and Tables S8 and S9, Supporting Information).

Our results suggest that human iPSC‐derived cortical organoids comprise multiple neuronal cell types, encompassing mature neurons and astrocytes.

### 2.5 Single‐Cell Transcriptomics Confirm Neuronal Loss and Astrocytosis in POLG Patient Cortical Organoids and Reveal Alterations in Multiple Regulatory Pathways

Next, we compared the single‐cell transcriptomic profiles between the patient and control samples. For the patient group, cortical organoids derived from compound heterozygous CP2A were utilized. We analyzed a total of 3331 cells from the patient organoid, which expressed 25 389 genes (Table S6, Supporting Information).

The patient cortical organoids of cell type enrichment analysis in scRNA‐seq analysis could be subcategorized further into GABAergic neurons (*H3F3B*, *NREP*, *SOX4*, *NR2F2*, *MLLT11*, *EIF4G2, RTN, RBFOX2, GAD1*), glutaminergic neurons (*TCF7L2*, *GAP43, STMN2*, *MAP1B*, *STMN1*, *SCG2*, *NEFL*, *STMN4*, *PGM2L1*), dopaminergic neurons (*RTN1*, *NREP*, *STMN2*, *PTMA*, *NSG2*, *TUBA1A*, *NOVA1*, *MLLT11*, *TMSB10*) and neural progenitor cells (*RPS19*, *RPS27*, *EEF1A1*, *RPL37*, *RPL7*, *RPLP0*, *RPL18A*, *C1orf61*, *RPL13A*). Melanocytes (*MLANA*, *PMEL*, *TYRP1*, *ANXA2*, *GPNMB*, *DCT*, *SAT1*, *QPCT*, *LGALS1*) were also detected, along with radial glial cell (*C1orf61*, *PTN*, *GPM6B*, *VIM*, *CLU*, *PTPRZ1*, *CNN3*, *TTYH1*, *EDNRB*), along with tanycytes (*SPARCL1*, *GPM6B*, *CLU*, *PTN*, *NTRK2*, *ATP1A2*, *PSAT1*, *SLC1A3*, *SPARC*), along with fibroblasts (*LUM*, *COL3A1*, *DCN*, *LGALS1*, *COL5A1*, *RPS18*, *RPL37*, *RPL31*, *RPL13*), ependymal cells (*CLU*, *SPARCL1*, *IFITM3*, *SPARC*, *B2M*, *RPS27L*, *SERF2*, *PLTP*, *MGST1*) and astrocyte‐related genes (*ATP1A2*, *NTRK2*, *SLC1A3*, *SPARCL1*, *GPM6B*, *PTN*, *ADGRG1*, *CLU*, *TTYH1*) (Figure 5G,H; Table S10, Supporting Information).

Using the UMAP annotation plot, we identified ten distinct cell population clusters (Figure 5E–H), identified through the expression of specific markers (Table S10, Supporting Information). The patient cortical organoid comprised 1.27% astrocytes, 25.82% dopaminergic neurons, 5.25% ependymal cells, 0.65% fibroblasts, 5.22% GABAergic neurons, 26.54% glutaminergic neurons, 4.16% melanocytes, 6.93% neural progenitor cells, 22.41% radial glial cells, and 1.74% tanycytes (Figure 5E; Figure S8, Supporting Information). Further classification of the neuronal populations revealed 40.03% dopaminergic neurons, 8.09% GABAergic neurons, 41.14% glutaminergic neurons, and 10.74% neural progenitor cells (Figure 5H).

Patient cortical organoids displayed neurons, radial glial cells, astrocytes, and ependymal cells, similar to controls. However, unique clusters in the patient cortical organoids included melanocytes, tanycytes and fibroblasts (Figure 5E,G,H). When compared to controls, there was a decrease in the percentage of neurons (from 83.54% in control to 64.51% in patient organoids), with an increase in astrocytes (from 0.02% in control to 1.27% in patient organoids) and radial glial cells (from 16.40% in control to 22.41% in patient organoids) (Figure 5G,H). A notable finding was the marked reduction of dopaminergic neurons and DA GLU neurons in patient cortical organoids (Figure 5G,H).

**Figure 6:**
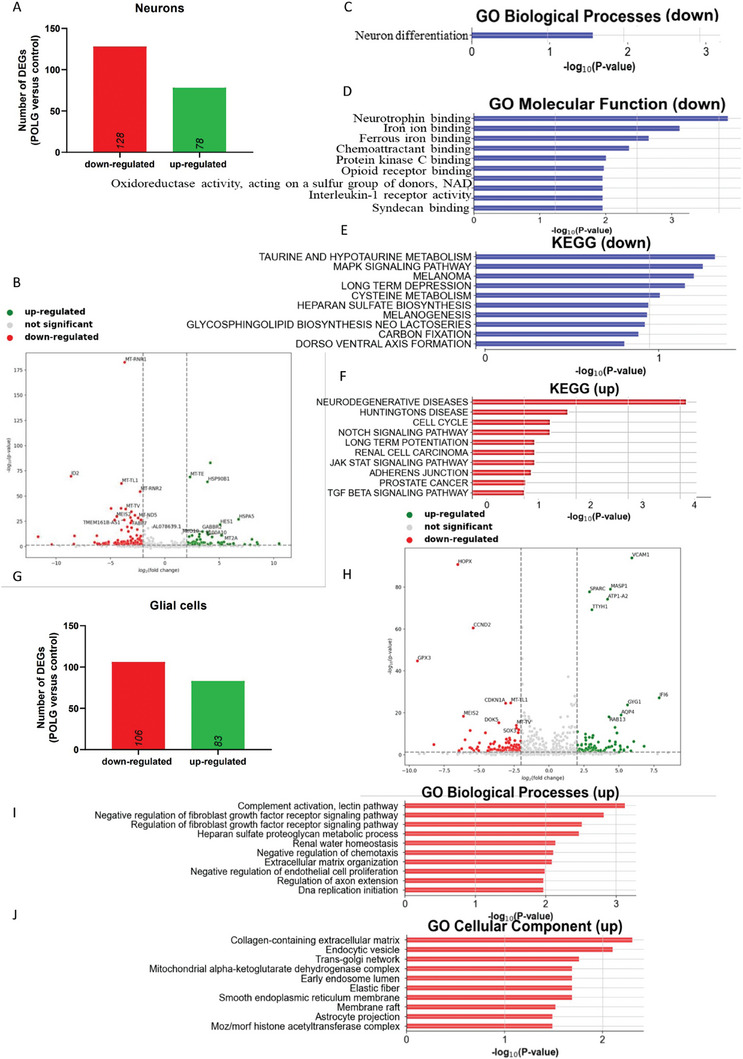
Comparison of molecular pathways in cortical organoids from control and patient iPSCs. A) The number of DEGs that are upregulated and downregulated in neuron population of patient CP2A cortical organoids compared to control cortical organoids. B) Volcano plot illustrating the DEGs in neuron population of patient CP2A cortical organoids compared to control cortical organoids. control cortical organoids C) GO biological progress enriches downregulated DEGs in neuron population of patient CP2A cortical organoids compared to control cortical organoids. patient cortical organoids control cortical organoids D) GO molecular functions enriched for downregulated DEGs in the neuron population of patient CP2A cortical organoids compared to control cortical organoids. control cortical organoids E) KEGG pathways enriched for downregulated DEGs in neuron population of patient CP2A cortical organoids compared to control cortical organoids. control cortical organoids F) KEGG pathways enriched for upregulated DEGs in neuron population of patient CP2A cortical organoids compared to control cortical organoids. control cortical organoids G) The number of DEGs that are upregulated and downregulated in glial cell population (astrocytes and radial glial cells) of patient CP2A cortical organoids compared to control cortical organoids. H) Volcano plot illustrating the DEGs in glial cell population (astrocytes and radial glial cells) in patient CP2A cortical organoids and control cortical organoids. I) GO biological processes enriched for upregulated DEGs in glial cell population (astrocytes and radial glial cells) in patient CP2A cortical organoids compared to control POLG CP2A‐isogenic control cortical organoids. J) GO cellular components enriched for upregulated DEGs in glial cell population (astrocytes and radial glial cells) in patient CP2A cortical organoids compared to control cortical organoids.

Gene expression analysis of neuronal populations in patients and control cortical organoids showed 206 differentially expressed genes (DEGs) in patients, with 78 being up‐regulated and 128 down‐regulated (**Figure** **6**A). Particularly, several mtDNA‐encoded genes, such as *MT‐ND5, MT‐RNR1, MT‐RNR2, MT‐TL1*, and *MT‐TY*, were among the most significantly downregulated genes (Figure 6B). Enrichment analysis for the downregulated genes highlighted functions relating to neuronal differentiation, dendrite formation, iron metabolism, reactive oxygen species, amino acid metabolism, and MAPK signaling (Figure 6C–E, **Tables** **1** and **2**; FiguresS9 and Table S11, Supporting Information). On the other hand, upregulated genes were enriched in pathways tied to neurodegenerative diseases, NOTCH and JAK‐STAT signaling pathways (Figure 6F and **Table** **3**).

**Table 1:** GO enrichment by down‐regulated DEGs in neuron population of patient organoids versus controls.

GO enrichment (down)	p‐value
Neuron differentiation	0.0330

GO molecular function (down)	p‐value
neurotrophin binding	0.0004
iron ion binding	0.0020
ferrous iron binding	0.0051
chemoattractant activity	0.0093
protein kinase C binding	0.0189
protease binding	0.0207
opioid receptor binding	0.0273
oxidoreductase activity, acting on a sulfur group of donors, NAD	0.0273
interleukin‐1 receptor activity	0.0274
syndecan binding	0.0274
testosterone dehydrogenase	0.0274
TPR domain binding	0.0274
prostaglandin E receptor activity	0.0274
3′,5′‐cyclic‐GMP phosphodiesterase activity	0.0378
ferric ion binding	0.0378
transmembrane receptor protein tyrosine kinase activity	0.0418
transmembrane receptor protein kinase activity	0.0418
G protein‐coupled receptor binding	0.0418
estradiol 17‐beta‐dehydrogenase activity	0.0421
type II transforming growth factor beta receptor binding	0.0424
cAMP response element binding protein binding	0.0473
histone methyltransferase activity	0.0530
interleukin‐1 receptor binding	0.0530
phosphate ion binding	0.0530
leucine zipper domain binding	0.0530
prostaglandin receptor activity	0.0530
protein tyrosine kinase binding	0.0578
AMP binding	0.0578
platelet‐derived growth factor binding	0.0578
MAP‐kinase scaffold activity	0.0578

**Table 2:** KEGG pathways enriched by down‐regulated DEGs in neuron population of patient organoids versus controls.

KEGG (down)	p‐value
Taurine and hypotaurine metabolism	0.0434
Mapk signaling pathway	0.0506
Melanoma	0.0573
Long term depression	0.0649
Cysteine metabolism	0.0900
Heparan sulfate biosynthesis	0.1039
Melanogenesis	0.1069
Glycosphingolipid biosynthesis neo lactoseries	0.1080
Carbon fixation	0.1188
Dorso ventral axis formation	0.1399
Thyroid cancer	0.1441
Cytokine cytokine receptor interaction	0.1590
Gluthatione metabolism	0.1897
Bladder cancer	0.2022
Lysine degradation	0.2238
Endometrial cancer	0.2441
Acute myeloid leukemia	0.2484
Androgen and estrogen metabolism	0.2527
Arachidonic acid metabolism	0.2505
Non small cell lung cancer	0.2520
Glycan structures biosynthesis 2	0.2838
Focal adhesion	0.2921
Glycolysis and gluconeogenesis	0.2921
Glioma	0.2921
Long term potentiation	0.3096
Renal cell carcinoma	0.3096
Vegf signaling pathway	0.3150
Pancreatic cancer	0.3252
Fc epsilon ri signaling pathway	0.3338
Chronic myeloid leukemia	0.3377

**Table 3:** KEGG pathways enriched by up‐regulated DEGs in neuron population of patient organoids versus controls.

KEGG (up)	p‐value
Neurodegenerative diseases	0.0001
Huntingtons disease	0.0147
Cell cycle	0.0315
Notch signaling pathway	0.0315
Long term potentiation	0.0629
Renal cell carcinoma	0.0629
JAK STAT signaling pathway	0.0629
Adherens junction	0.0754
Prostate cancer	0.0905
TGF beta signaling pathway	0.0973
Melanogenesis	0.1255
Alzheimers disease	0.1506
Basal transcription factors	0.1943
MAPK signaling pathway	0.2017
Sphingolipid metabolism	0.2123
N glycan biosynthesis	0.2235
WNT signaling pathway	0.2235
Inositol phosphate metabolism	0.2662
Tryptophan metabolism	0.3074
B cell receptor signaling pathway	0.3236
Epithelial cell signaling in helicobacter pylori infection	0.3407
Phosphatidylinositol signaling system	0.3777
Colorectal cancer	0.4058
ERBB signaling pathway	0.4185
T cell receptor signaling pathway	0.4361
Toll like receptor signaling pathway	0.4591

**Figure 7:**
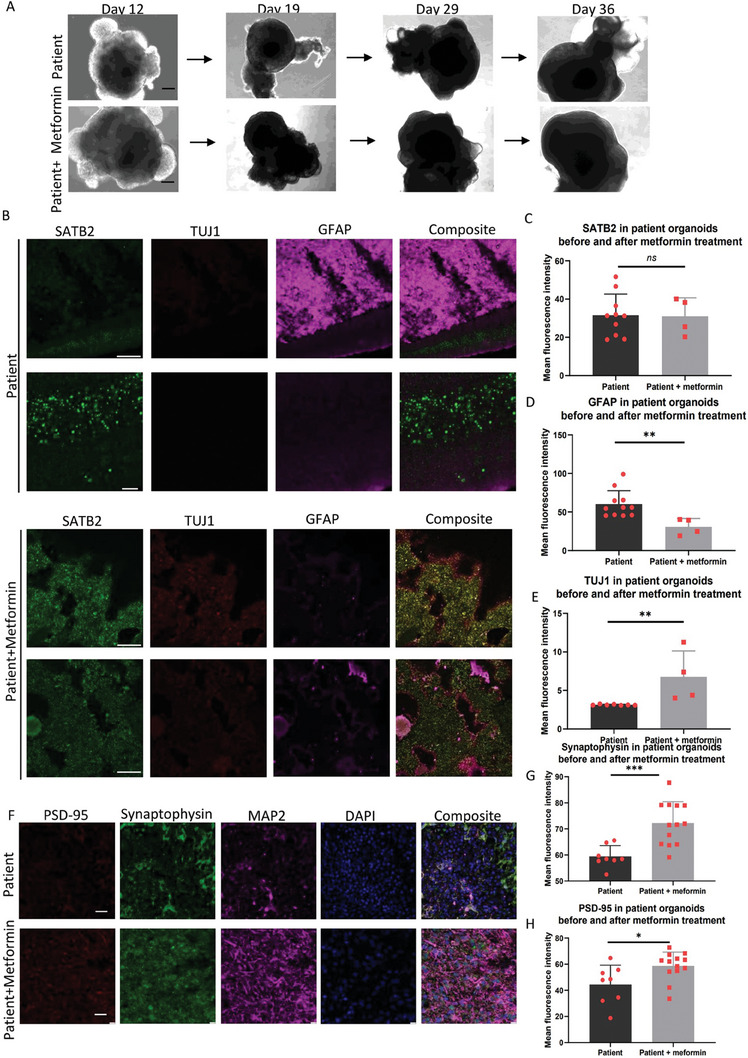
Comparison of lineage markers in patient brain organoid before and after metformin treatment. A) Representative phase contrast images of the cell morphology of patient WS5A brain organoids before and after metformin treatment at day 12, 19, 29, and 36. Black scale bar is 100 µm. B) Representative immunofluorescent imaging of cryo‐sectioned organoids at 36 days, staining the cortical pyramidal neuronal marker SATB2 (green), newborn neural marker TUJ1 (green), and reactivated astrocyte marker GFAP (red) in patient WS5A cortical organoids and patient WS5A cortical organoids treated with metformin. Scale bar is 100 µm. C–E) Quantitative measurements of the level of SATB2 D), GFAP E), and TUJ1 F) expression on day 36 in patient cortical organoids and patient cortical organoids treated with metformin. The Y‐axis represents the mean fluorescence intensity The data were presented as mean ± SD. Significance levels are indicated for P values less than 0.05, with ** indicating *P* < 0.01, *** indicating *P* < 0.001, **** indicating *P* < 0.000. Results not reaching statistical significance are denoted as ns (not significant). The information on sample size (n), probability (P) value, and the specific statistical test for each experiment was presented in Tables S5 and S9 (Supporting Information). F) Representative immunofluorescent imaging of cryo‐sectioned organoids, staining the presynaptic marker Synaptophysin (green), postsynaptic marker PSD‐95 (red), and mature neural marker MAP2 (purple) in patient WS5A cortical organoids and patient WS5A cortical organoids treated with metformin. Nuclei are stained with DAPI (blue). Scale bar is 100 µm. G,H) Quantitative measurements of the level of Synaptophysin G) and PSD‐95 H) expression in patient cortical organoids and patient cortical organoids treated with metformin. The Y‐axis represents the mean fluorescence intensity. The data were presented as mean ± SD. Significance levels are indicated for P values less than 0.05, with * indicating *P* < 0.05, ** indicating *P* < 0.01, *** indicating *P* < 0.001, **** indicating *P* < 0.000. Results not reaching statistical significance are denoted as ns (not significant). The information on sample size (n), probability (P) value, and the specific statistical test for each experiment was presented in Tables S5 and S9 (Supporting Information).

Looking at the glial cell population, consisting of radial glial cells and astrocytes, we found 189 DEGs when compared to the control cortical organoids, with 83 genes upregulated and 106 genes downregulated in the patient organoids' glial population (**Figure** **7**G,H). GO analysis on the regulated genes in this population revealed an enrichment in processes including complement activation pathway and astrocyte projection in the patient samples as opposed to controls (Figure 7I,J and **Table** **4**, **5**). Conversely, downregulated genes in glial cells were enriched in processes like central nervous system development, axon guidance and nervous system development (Figure S10 and Table S12, Supporting Information).

**Table 4:** GO enrichment and molecular functions of up‐regulated DEGs in glial cell population of patient organoids versus controls.

GO biological process (up)	p‐value
Complement activation, lectin pathway	0.0007
Negative regulation of fibroblast growth factor receptor signaling pathway	0.0015
Regulation of fibroblast growth factor receptor signaling pathway	0.0031
Heparan sulfate proteoglycan metabolic process	0.0034
Renal water homeostasis	0.0072
Negative regulation of chemotaxis	0.0078
Extracellular matrix organization	0.0082
Negative regulation of endothelial cell proliferation	0.0104
Regulation of axon extension	0.0110
Dna replication initiation	0.0110
Bone development	0.0120
Extracellular structure organization	0.0124
External encapsulating structure organization	0.0127
Positive regulation of axonogenesis	0.0136
Cellular response to interferon‐gamma	0.0141
Regulation of respiratory gaseous exchange by nervous system process	0.0206
Gastro‐intestinal system smooth muscle contraction	0.0209
Glial cell‐derived neurotrophic factor receptor signaling pathway	0.0209
Axon choice point recognition	0.0209
Schwann cell differentiation	0.0209
Negative regulation of synapse organization	0.0209
Protein‐dna complex assembly	0.0221
Cellular water homeostasis	0.0248
Negative regulation pf amyloid precursor protein biosynthetic process	0.0250
Protein localization to non‐motile cilium	0.0250
Mrna 5'‐splice site recognition	0.0250
Chondrocyte development	0.0251
Macromolecule biosynthetic process	0.0252
Melanosome assembly	0.0252
Retinal ganglion cell axon guidance	0.0252

**Table 5:** GO cellular component of up‐regulated DEGs in glial cell population of patient organoids versus controls.

GO cellular component (up)	p‐value
Collagen‐containing extracellular matrix	0.0050
Endocytic vesicle	0.0080
Trans‐golgi network	0.0175
Mitochondrial alpha‐ketoglutarate dehydrogenase complex	0.0205
Early endosome lumen	0.0205
Elastic fiber	0.0207
Smooth endoplasmic reticulum membrane	0.0207
Membrane raft	0.0304
Astrocyte projection	0.0330
Moz/morf histone acetyltransferase complex	0.0330
Insulim‐responsive compartment	0.0367
H3 histone acetyltransferase complex	0.0367
Multimeric ribonuclease p complex	0.0411
Sodium:potassium‐exchanging atpase complex	0.0411
Transcription factor tfiih core complex	0.0411
Microfibril	0.0446
Melanosome membrane	0.0489
Chitosome	0.0489
Pigment granule membrane	0.0489
Transcription factor tfiih holo complex	0.0489
Lysosomal lumen	0.0498
Beta‐catenin‐tcf complex	0.0530
Podosome	0.0533
Mhc class ii protein complex	0.0533
Prespliceosome	0.0609
Trans‐golgi network transport vesicle	0.0615
U2‐type prespliceosome	0.0615
Cation‐transporting atpase complex	0.0650
Platelet alpha granule membrane	0.0684
U1 snrnp	0.0723

In the cohort of dopaminergic neurons, analysis revealed 115 DEGs that were upregulated and 29 that were downregulated in patient cortical organoids compared to those that were control (Figure S11, Supporting Information). An enrichment analysis of the downregulated DEGs within this dopaminergic neuron population showed an association with several GO molecular function processes and cellular components. Notably, these included NAD^+^ ADP‐ribosyltransferase activity within molecular functions, while cellular components encompassed structures such as the apical dendrite, dendrite, microtubule and cytoskeleton, spindle and spindle microtubule, asymmetric synapse, postsynaptic density, cytoskeleton, and axon (Figures S12 and S13 and Tables S13 and S14, Supporting Information). A KEGG pathway analysis of the downregulated DEGs within this dopaminergic neuron population showed enriched WNT, JAK STAT and MARK signaling pathways (Figure S14, Supporting Information).

Overall, these findings demonstrate substantial differences in gene expression between patient and control cortical organoids, revealing significant changes in key neuronal and glial cell populations. The dysregulation of numerous genes related to neuronal differentiation, central nervous system development signaling pathways, in conjunction with shifts in the composition of specific neuronal cell types, underscores the complex genetic and cellular landscape of POLG‐related disease.

### 2.6 Metformin Ameliorates the Phenotype of the POLG Cortical Organoid

In the realm of therapeutic interventions for POLG‐associated disorders, the role of metformin as a treatment option has garnered considerable attention. Building on our previous work, we've substantiated its effectiveness through an array of tests.^[^ ^26^ ^]^ A closer examination of metformin's potential therapeutic pathways reveals its intimate association with mitochondrial operations and cellular metabolic processes. This connection, in the milieu of POLG‐related diseases, might be instrumental in delivering its curative properties. We next explored the potential of our organoid as a platform for preclinical drug trials. According to prior studies, metformin could alleviate neuronal damage/loss, encourage neuronal differentiation, and suppress astrocyte formation in a cerebral ischemia/reperfusion rat model.^[^ ^23^ ^]^ We, along with others, have found that metformin fosters mitochondrial biogenesis and bolsters mitochondrial activity and function.^[^ ^26^, ^28^, ^29^, ^30^ ^]^ Thus, we asked if metformin could mitigate the phenotype and molecular profile of the POLG‐organoid. We exposed patient cortical organoids to 250 µm metformin from day 6 and observed an enhancement in cortical organoid structure and morphology after a 30‐day (Figure 7A). Moreover, metformin‐treated organoids showed reduced expression of astrocytic marker GFAP (Figure 7B,D) and elevated levels of neuronal marker TUJ1 (Figure 7B,E), compared to their untreated counterparts, while the cortical neural marker SATB2 remained stable (Figure 7B,C). We also noted an upswing in both synaptophysin (Figure 7F,G), and PSD‐95 expressions (Figure 7F,H) compared to the untreated samples.

**Figure 8:**
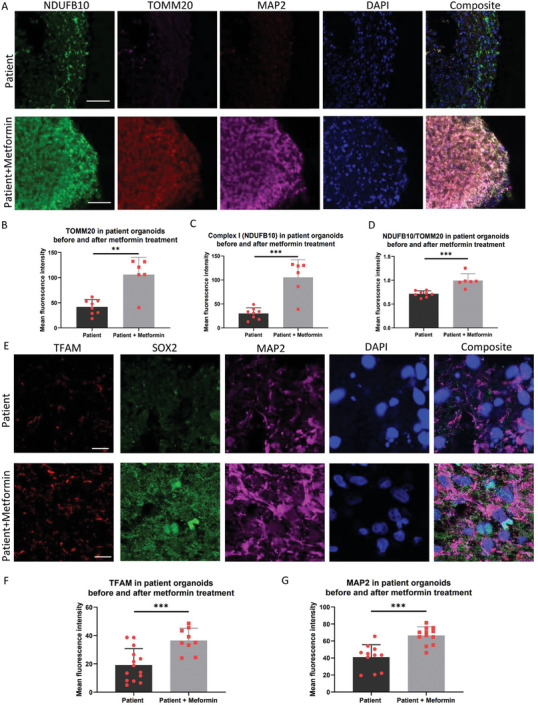
Metformin rescues the neuronal damage and mitochondrial defect in POLG patient brain organoid. A) Representative immunofluorescent imaging of cryo‐sectioned organoids at 90 days, showing the staining of NDUFB10 (green), TOMM20 (red), and MAP2 (purple) in patient WS5A cortical organoids and patient WS5A cortical organoids treated with metformin. Nuclei are stained with DAPI (blue). Scale bar is 100 µm. B–D) Quantitative measurements of the level of B) TOMM20, C) NDUFB10, and D) NDUFB10/TOMM20 expression in patient cortical organoids and patient treated with metformin. The Y‐axis represents the mean fluorescence intensity. The data were presented as mean ± SD. Significance levels are indicated for P values less than 0.05, with** indicating P < 0.01, *** indicating P < 0.001. The information on sample size (n), probability (P) value, the specific statistical test for each experiment was presented in Tables S5 and S9 (Supporting Information). E) Representative imaging of cryo‐sectioned organoids at 36 days, staining TFAM (red), SOX2 (green), and MAP2 (purple) in patient WS5A cortical organoids and patient WS5A cortical organoids treated with metformin. Nuclei are stained with DAPI (blue). Scale bar is 100 µm. F,G) Quantitative measurements of the level of TFAM F) and MAP2 G)in patient cortical organoids and patient cortical organoids treated with metformin. The Y‐axis represents the mean fluorescence intensity. The data were presented as mean ± SD. Significance levels are indicated for P values less than 0.05, with * indicating P < 0.05, ** indicating P < 0.01, *** indicating P < 0.001. The information on sample size (n), probability (P) value, and the specific statistical test for each experiment was presented in Tables S5 and S9 (Supporting Information).

**Figure 9:**
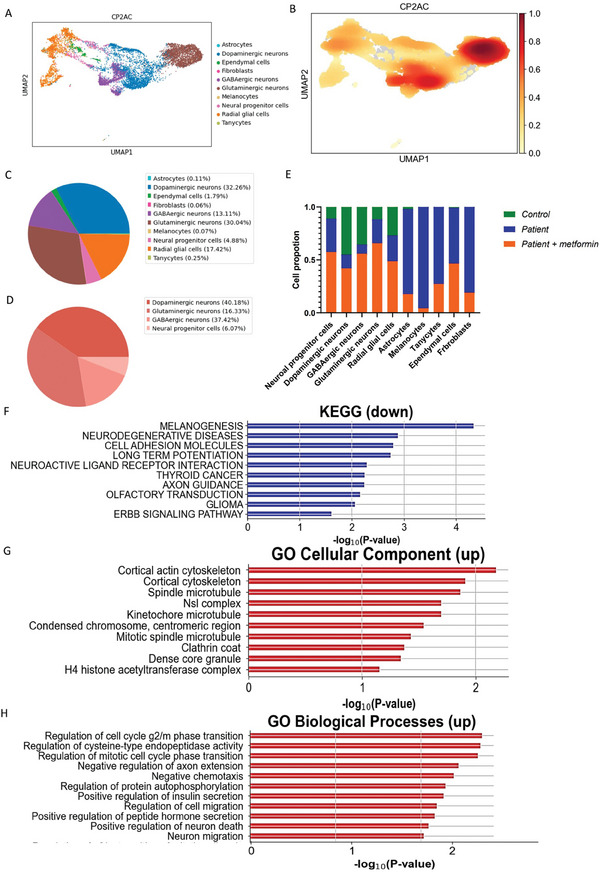
Comparison of molecular pathways in cortical organoids from patient brain organoid before and after metformin treatment. A,B) Cell clusters in 3‐month‐old organoids derived from patient CP2A iPSCs with metformin treatment were visualized using the scMRMA and UMAP algorithm A), and their density is depicted in B). C,D) The percentage distribution of all cell clusters C) and the neuro population D) in patient CP2A cortical organoids with metformin treatment, were represented with the PCA algorithm. E) Percentage histogram showing the distribution of individual cell clusters in control cortical organoids, patient CP2A cortical organoids, and patient cortical organoids treated with metformin. F) KEGG pathways enriched for down‐regulated differentially expressed genes (DEGs) in the neuron population of patient CP2A cortical organoids compared to patient CP2A cortical organoids treated with metformin. G) GO cellular components enriched for up‐regulated DEGs in the neuron population of patient CP2A cortical organoids compared to patient CP2A cortical organoids treated with metformin. H) GO biological processes enriched for up‐regulated DEGs in the neuron population of patient CP2A cortical organoids compared to patient CP2A cortical organoids treated with metformin.

When we stained complex I (NDUFB10), TOMM20 and MAP2, we detected a marked increase in the levels of TOMM20 (**Figure** **8**A,B), MAP2 (Figure 8A,E,G; Figure S15, Supporting Information) and total complex I (Figure 8A,C; Figure S15, Supporting Information) in metformin treated samples compared to untreated ones. On normalizing total complex I (NDUFB10) to the mitochondrial mass (TOMM20), we saw a significantly higher level of NDUFB10/TOMM20 in metformin treated patient cortical organoids (Figure 8A,D). Further, immunostaining of TFAM, SOX2 and MAP2 showed significantly increased levels of TFAM (**Figure** **9**E,F) and MAP2 expression (Figure 8E,G) in the patient cortical organoids suggesting an increase in mtDNA copy number and neural regrowth with metformin treatment compared to untreated samples.

In summary, metformin treatment of POLG organoids led to improved structure and morphology, an increased neuronal marker and reduced astrocytic markers, increased mitochondrial mass and complex I and TFAM expression. These results demonstrate metformin's potential for mitigating the phenotype and enhancing the molecular profile of POLG organoids, validating the utility of organoid models in preclinical drug trials.

### 2.7 Single‐Cell Transcriptomics Confirm Neuronal Enrichment and Decreased Astrocytosis in POLG Organoids upon Metformin Treatment

We next evaluated the impact of metformin on the single‐cell transcriptomic profile of POLG organoids by treating the CP2A patient cortical organoids for over 2 months. We identified a total of 8433 cells expressing 26403 genes (Table S6, Supporting Information). The patient cortical organoids upon metformin treatment of cell type enrichment analysis in scRNA‐seq analysis could be subcategorized further into dopaminergic neurons (MAB21L1, *PTMA*, *RTN1*, *TMSB10*, *MLLT11*, *NREP*, *STMN2*, *H3F3B*, *VSNL1*), ependymal cells (*CLU*, *IFITM3*, *SPARC*, *SPARCL1*, *B2M*, *NPC2*, *SERF2*, *GSTP1*, *CRYAB*), along with fibroblasts (*COL3A1*, *COL1A2*, *COL1A1*, *LGALS1*, *ISLR*, *COL6A3*, *LUM*, *MFAP4*, *POSTN*), GABAergic neurons (*SOX4*, *LHX1*, *NREP*, *ZNF385D*, *H3F3B*, *TFAP2A*, *RTN1*, *BLCAP*, *ETFB*), glutaminergic neurons (*TCF7L2*, *NEFL*, *STMN2*, *GAP43*, *MAP1B*, *STMN1*, *LHX9*, *NEFM*, *WLS*), melanocytes (*TYR*, *TYRP1*, *DCT*, *MLANA*, *PMEL*, *LGALS3*, *PTGDS*, *SAT1*, *CTSB*) and neural progenitor cells (*RPS27L*, *RPS27*, *RPS19*, *FTL*, *GNG5*, *RPLP1*, *RPL7*, *VIM*, *RPS3A*) were also detected, along with radial glial cells (*PTN*, *C1orf61*, *VIM*, *GPM6B*, *GNG5*, *DBI*, *SPARC*, *CNN3*, *TTYH1*), along with tanycytes (*SPARCL1*, *ATP1A2*, *PTN*, *CLU*, *NTRK2*, *MGST1*, *IFITM3*, *PLTP*, *VIM*), and astrocyte‐related genes (*ATP1A2*, *SPARCL1*, *SLC1A3*, *PI15*, *QKI*, *PTPRZ1*, *GPM6B*, *PTN*, *NTRK2*) (**Fig** **ure** 9A–D).

The UMAP plot identified eight distinct cell clusters, which included neurons (80.29%), two subtypes of radial glial cells (17.42%), astrocytes (0.11%), ependymal cells (1.79%), melanocytes (0.07%), tanycytes (0.25%) and fibroblasts (0.06%) (Figure 9A–D; Figure S16, Supporting Information). Interestingly, even after metformin treatment, we did not detect a DA GLU neuron population in the POLG organoids, indicating that metformin may not alleviate their absence (Figure 9A–D; Table S15, Supporting Information).

Further comparison of cell cluster proportions between control cortical organoids, untreated POLG organoids, and metformin‐treated POLG organoids indicated shifts in cell populations. Metformin treatment appeared to reduce the astrocyte population while increasing the neuron population when we compared untreated and treated POLG organoids (Figure 9E). Furthermore, metformin seemed to decrease the presence of melanocytes, tanycytes, and fibroblasts (Figure 9E).

Pathway analysis in the neuronal population revealed downregulated genes were enriched for multiple pathways, including those associated with neurodegenerative diseases and axon guidance (Figure 9E, **Table** **6**). On the other hand, upregulated genes were enriched for processes related to cortical actin cytoskeleton, cortical cytoskeleton, neuronal migration, and regulation of neurotransmitter transport (Figure 9F,G, **Tables** **7** and **8**; Figure S17, Supporting Information). Similar patterns were observed in glial cells, where downregulated genes showed enrichment for pathways related to neurodegenerative diseases (Figure S18 and Table S17, Supporting Information).

**Table 6:** KEGG pathways of down‐regulated DEGs in the neuron population in treated patient organoids versus untreated group.

KEGG (down)	p‐value
Melanogenesis	0.0000
Neurodegenerative diseases	0.0013
Cell adhesion molecules	0.0017
Long term potentiation	0.0020
Neuroactive ligand receptor interaction	0.0052
Thyroid cancer	0.0060
Axon guidance	0.0061
Olfactory transduction	0.0070
Glioma	0.0086
ERBB signaling pathway	0.0253
Prostate cancer	0.0260
Endometrial cancer	0.0297
Acute myeloid leukemia	0.0305
Basal cell carcinoma	0.0339
GNRH signaling pathway	0.0358
GAP junction	0.0368
WNT signaling pathway	0.0409
Alzheimers disease	0.0494
Renal cell carcinoma	0.0565
Huntingtons disease	0.0565
Adherens junction	0.0701
Long term depression	0.0720
Fatty acid biosynthesis	0.0759
Insulin signaling pathway	0.0892
Bladder cancer	0.1048
MAPK signaling pathway	0.1106
Non small cell lung cancer	0.1486
VEGF signaling pathway	0.2164
Melanoma	0.2346
FC Epsilon RI signaling pathway	0.2475

**Table 7:** GO cellular component of up‐regulated DEGs in the neuron population in treated patient organoids versus untreated group.

GO cellular component (up)	p‐value
Cortical actin cytoskeleton	0.0065
Cortical cytoskeleton	0.0122
Spindle microtubule	0.0135
Nsl complex	0.0198
Kinetochore microtubule	0.0198
Condensed chromosome, centromeric region	0.0286
Mitotic spindle microtubule	0.0375
Clathrin coat	0.0426
Dense core granule	0.0445
H4 histone acetyltransferase complex	0.0672
Nuclear inner membrane	0.0785
Condensed chromosome	0.1407
Tertiary granule lumen	0.1468
Caveola	0.1576
Specific granule lumen	0.1645
Clathrin‐coated endocytic vesicle membrane	0.1791
Cell‐cell junction	0.1817
Voltage‐gated potassium channel complex	0.1869
Endoplasmic reticulum lumen	0.1978
Lipid droplet	0.2035
Potassium channel complex	0.2064
Plasma membrane raft	0.2094
Clathrin‐coated endocytic vesicle	0.2154
Clathrin‐coated vesicle membrane	0.2248
Azurophil granule lumen	0.2280
Actin cytoskeleton	0.2313
Secretory granule lumen	0.2313
Intracellular non‐membrane‐bounded organelle	0.2414
Integral component of plasma membrane	0.2483
Microtubule cytoskeleton	0.2448

**Table 8:** GO biological processes of up‐regulated DEGs in the neuron population in treated patient organoids versus untreated group.

GO biological processes (up)	p‐value
Regulation of cell cycle g2/m phase transition	0.0020
Regulation of cysteine‐type endopeptidase activity	0.0021
Regulation of mitotic cell cycle phase transition	0.0022
Negative regulation of axon extension	0.0036
Negative chemotaxis	0.0042
Regulation of protein autophosphorylation	0.0052
Positive regulation of insulin secretion	0.0056
Regulation of cell migration	0.0066
Positive regulation of peptide hormone secretion	0.0071
Positive regulation of neuron death	0.0081
Neuron migration	0.0094
Regulation of g2/m transition of mitotic cell cycle	0.0094
Regulation of cytokine production	0.0100
Heart morphogenesis	0.0106
Negative regulation of viral genome replication	0.0106
Positive regulation of interleukin‐1 beta production	0.0119
Positive regulation of interleukin‐1 production	0.0139
Negative regulation of endopeptidase activity	0.0139
Negative regulation of metallopeptidase activity	0.0139
Cellular response to heparin	0.0147
Lipid transport across blood‐brain barrier	0.0147
Positive regulation of amino acid transport	0.0150
Cellular response to type I interferon	0.0150
Type I interferon signaling pathway	0.0150
Regulation of viral genome replication	0.0166
Histamine metabolic process	0.0172
Negative regulation of amino acid transport	0.0172
Regulation of macromolecule biosynthetic process	0.0176
Regulation of hormone biosynthetic process	0.0176
Positive regulation of tau‐protein kinase activity	0.0176

In the population of dopaminergic neurons, a total of 39 DEGs were identified as upregulated, while 251 were downregulated in cortical organoids from patients treated with metformin, as compared to untreated organoids (Figure S18, Supporting Information). When analyzing the upregulated DEGs within the Dopaminergic neuron population, several GO cellular components were found to be enriched. These included the integral and intrinsic component of mitochondrial outer membrane, integral component of mitochondrial membrane, mitochondrial outer membrane and synaptic membrane (Figure S20 and Table S18, Supporting Information). KEGG analysis showed the upregulation of MARK and MTOR signaling pathways (Figure S21, Supporting Information).

Our single‐cell transcriptomic analysis of metformin treated POLG organoids revealed changes in the proportions of different cell types and shifts in gene expression profiles, particularly in neuronal and glial populations, suggesting that metformin can influence neurodegenerative disease pathways and cellular components.

## 3 Discussion

In our study, we generated a detailed cellular and molecular profile of cortical organoids developed from two patient harboring known pathogenic *POLG* mutations and several healthy controls (**Table** **9**). The organoids recapitulated key aspects of the disease process, providing insights into disease mechanisms and a robust platform for drug testing. Immunostaining and scRNA‐seq in our novel, human derived organoid model of POLG encephalopathy confirmed the presence of multiple types of neuronal and glial cells, consistent with differentiation into brain‐like tissue.^[^ ^31^ ^]^ The scRNA‐seq data also indicated the presence of radial glial cells, however, indicating that the maturation of the organoids is not complete. This is a common technical limitation of brain organoid research.^[^ ^14^, ^32^ ^]^ Nevertheless, our model reliably recapitulated important hallmark tissue and molecular changes we have observed in our postmortem studies of patients^[^ ^24^, ^31^ ^]^ with POLG encephalopathy, including neuronal loss affecting both dopaminergic and DA GLU neuron populations, astrocytosis, complex I deficiency and mtDNA depletion.

**Table 9:** List of the demographic information of samples used in the study.

iPSC line	Mutation	Age	Gender	Source
Control Detroit 551	no	fetal	female	ATCC® CCL 110™
Control CCD‐1079Sk	no	new‐born	male	ATCC® CRL‐2097™
Control AG05836	no	44 yrs	female	RRID:CVCL2B58
POLG WS5A‐isogenic control	no	45 yrs	female	gene corrected‐POLG WS5A patient iPSC
POLG WS5A	homozygous c.2243G>C, p.W748S/W748S	45 yrs	female	POLG WS5A patient fibroblast
POLG CP2A	compound heterozygous c.1399G>A/c.2243G>C, p.A467T/W748S	50 yrs	male	POLG CP2A patient fibroblast

Our results demonstrated successful generation of cortical organoids from iPSCs using a neural induction protocol involving dual SMAD inhibition and canonical Wnt inhibition. The organoids exhibited progressive development, with the appearance of neural identity markers and the formation of cortical brain regions. At different stages of development, we observed distinct characteristics in the organoids, including the expression of neural markers and the presence of different neuronal cell types. By day 90, the organoids consisted predominantly of mature neurons positive for the marker NeuN, along with smaller populations of cells expressing GFAP, MAP2, and SOX2. Furthermore, the cortical pyramidal neuronal markers SATB2 and CTIP2 were observed in specific layers of the organoids, and stratified expressions of oligodendrocyte marker OLIG2, astrocyte marker GFAP, and neural marker DCX were detected. Importantly, the patient‐derived organoids exhibited significant structural alterations compared to control cortical organoids, characterized by neuronal loss, and increased astrocytosis. The patient cortical organoids showed irregular neuroepithelial tissue organization, reduced ventricle‐like structures, and diminished expression of cortical neuron markers SATB2 and TUJ1. In contrast, there was a notable elevation in the expression of the activated astrocyte marker GFAP. These findings suggest that *POLG* mutations contribute to aberrant growth and altered neuronal development in cortical organoids.

To gain insights into the cellular composition and gene expression dynamics of the cortical organoids, we performed scRNA‐seq analysis. Our scRNA‐seq analysis revealed eight distinct cell population clusters in the control cortical organoids, including astrocytes, dopaminergic neurons, glutaminergic neurons, GABAergic neurons, neural progenitor cells, ependymal cells, radial glial cells, and DA GLU neurons. The DA GLU neurons were further classified into subpopulations based on gene expression profiles. Pathway enrichment analyses on the neuronal population confirmed the enrichment of gene sets associated with neural development processes, such as axon development, neural migration, generation of neurons, and synaptic transmission. Results from both immunostaining and scRNA‐seq revealed changes in cell type number and composition in cortical organoids from patients compared to healthy controls. Neuronal loss is one of the most severe phenotypic manifestations in the spectrum of diseases with *POLG* mutations^[^ ^31^, ^33^ ^]^ and we found significant neuronal loss in patient cortical organoids, including cortical neurons and ‐DA GLU neurons.

Our study not only revealed the loss of neurons but also highlighted the presence of astrocyte activation and reactive gliosis in patient‐derived organoids. The observed astrocyte activation is particularly significant as it has been shown in our study^[^ ^26^ ^]^ and previous research^[^ ^34^, ^35^, ^36^ ^]^ to be associated with neuronal toxicity and may contribute to the neural loss observed in POLG‐related disorders. Astrocytes play crucial roles in supporting neuronal function and maintaining brain homeostasis. However, under pathological conditions, such as *POLG* mutations, astrocytes can become activated and undergo reactive gliosis. This reactive response is characterized by changes in astrocyte morphology, gene expression, and secretion of various signaling molecules. The activation of astrocytes in POLG patient cortical organoids suggests a potential role for these cells in contributing to the disease pathology. Studies have demonstrated that activated astrocytes can release pro‐inflammatory factors, reactive oxygen species, and other neurotoxic substances, which can lead to neuronal dysfunction and degeneration. These toxic effects on neurons may contribute to the observed neuronal loss in diseases with *POLG* mutations. Moreover, the presence of reactive gliosis in patient‐derived organoids further supports the idea that these iPSC‐derived brain models can recapitulate key pathological features of diseases with *POLG* mutations. The activation of astrocytes and the subsequent gliosis response are hallmarks of various neurodegenerative disorders, including POLG‐related disorders. This similarity in pathological features between patient‐derived organoids and actual disease conditions strengthens the validity and relevance of these organoid models for studying diseases with *POLG* mutations mechanisms. By recapitulating the pathological changes observed in patient brains, iPSC‐derived brain organoids offer a valuable platform for investigating the underlying mechanisms of diseases with *POLG* mutations. These models provide an opportunity to explore the interactions between different cell types, such as neurons and astrocytes, and the impact of these interactions on disease progression.^[^ ^37^, ^38^, ^39^ ^]^ Furthermore, they enable the testing of potential therapeutic strategies aimed at modulating astrocyte activation and mitigating the neurotoxic effects associated with POLG‐related disorders.

In our study, we made an intriguing observation that patient cortical organoids with *POLG* mutations exhibited a loss of both neurons and markers associated with mature neuronal identity. Neurons are typically post‐mitotic cells that form during embryonic development and maintain their specialized characteristics, functionality, and resilience throughout an individual's lifespan. However, in certain circumstances, cell dedifferentiation can occur during developmental processes or in response to stress or injury.^[^ ^40^, ^41^, ^42^, ^43^, ^45^ ^]^ Recent evidence also suggests that loss of cellular identity is associated with neuronal vulnerability and neurogenesis.^[^ ^1^ ^]^ The loss of mature neuronal markers suggests that diseases with *POLG* mutations neurons may dedifferentiate, thereby reactivating the developing neural cell cycle. Evidence from familial AD‐iPSC‐derived neurons and postmortem AD brains also supports this phenomenon.^[^ ^45^ ^]^ Additionally, our findings indicate that patient cortical organoids exhibited a loss of postsynaptic markers, although the specific marker is not mentioned in the provided text. In vivo studies have revealed that postsynaptic cells play a critical role in initiating presynaptic differentiation and stabilizing immature synaptic contacts, enabling them to mature and respond to signals that promote synaptic maturation. Thus, our findings suggest that diseases with *POLG* mutations either leads to blocked postsynaptic differentiation,^[^ ^46^ ^]^ or that postsynaptic markers are lost as part of the dedifferentiation process, while presynaptic markers remain.

At the transcriptomic level, scRNA‐seq highlighted cell‐type‐specific variations in POLG organoids. In neuronal populations, we observed a notable downregulation of pathways tied to neuronal differentiation. This observation supports our results showing loss of mature neuronal markers that suggested a state of dedifferentiation. Loss of established neuronal identity and reversion to a more progenitor‐like state has, moreover, been observed in other neurodegenerative diseases.^[^ ^47^ ^]^ Intriguingly, we noticed upregulation of the NOTCH and JAK‐STAT signaling pathways. The NOTCH signaling pathway plays a crucial role in maintaining neural progenitor cells and inhibiting neuronal differentiation, which aligns with our observation of impaired neuronal maturation.^[^ ^48^ ^]^ The JAK‐STAT pathway is primarily associated with cellular responses to cytokines and growth factors and has been implicated in neuroinflammatory responses and glial cell activation.^[^ ^48^ ^]^ This upregulation could potentially reflect the observed astrocytosis in our model, indicating a response to neuronal damage or that astrocytes are playing a more active role in the disease process. These results exemplify the complex, multifaceted cellular changes resulting from *POLG* mutations and the potential interplay between genetic changes, cellular differentiation status, and intercellular signaling in disease progression.

We assayed the mitochondrial consequences of *POLG* mutations in “living organoid cells” and observed loss of complex I and mtDNA copies, consistent with our findings in patient postmortem brain tissue^[^ ^7^ ^]^ and 2D neuronal cell models.^[^ ^12^, ^13^ ^]^ The simultaneous loss of neurons in patient cortical organoids made it attractive to postulate a causal relationship between complex I loss and neural cell death,^[^ ^49^ ^]^ however, complex I loss was found to affect the whole brain in PD, not just the substantia nigra,^[^ ^50^, ^51^ ^]^ suggesting that loss of this complex may not itself be the primary cause of neuronal loss, but a compensatory response. Irrespective of whether neuronal loss of complex I a pathological event or compensatory response is, this feature is clearly important and associated with the diseases with POLG mutations process in both in patients and our cortical organoids. Following changes in level of both complex I and mtDNA may, therefore, be useful for monitoring treatment or other interventions.

Another key molecular consequence of *POLG* mutations is the presence of mtDNA depletion.^[^ ^31^, ^52^ ^]^ We have shown that this is present in neurons from infants under 1 year of age and a stable finding in surviving neurons of patients from all age groups.^[^ ^31^ ^]^ Here, using the patient cortical organoids, we also observed lowered mtDNA levels using an indirect method based on flow cytometric measurement of TFAM. While this method relies on an indirect assessment, TFAM binds mtDNA in molar quantities and we suggest that it can be used in live cells as a surrogate measure of mtDNA level.^[^ ^13^, ^25^ ^]^ The presence of mtDNA depletion appear to impair cell growth, as in the case of patient.^[^ ^24^, ^31^ ^]^ The presence of mtDNA depletion has important implications for cellular function and growth. In the case of the patient we studied, impaired cell growth was observed, which is consistent with previous reports linking mtDNA depletion to cellular dysfunction and compromised cell viability.^[^ ^53^ ^]^ The reduction in mtDNA levels may disrupt mitochondrial function, as mtDNA encodes essential genes involved in oxidative phosphorylation and ATP production. This impairment in energy production can negatively impact cell growth and survival. Efforts to increase mtDNA levels in cells with *POLG* mutations have been explored as a potential therapeutic strategy. One approach involves the use of mitochondrial‐targeted nucleotides or nucleotide analogs to enhance mtDNA replication and restore mtDNA levels.^[^ ^54^ ^]^ These compounds can bypass the impaired mtDNA replication machinery caused by *POLG* mutations and promote the synthesis of new mtDNA copies. However, it should be noted that the development of effective and safe therapies targeting mtDNA depletion in POLG‐related disorders is still in the early stages and requires further investigation.

Our results indicate that the POLG organoid model is suitable as a platform for drug testing and screening. Parameters such as cellularity and cell type complements, complex I quantity, mitochondrial mass as measured by TOMM20 quantity, and mtDNA copy number measured indirectly by assessing TFAM, can serve as sensitive readouts to assess drug effects. As proof of concept, we show that exposure to metformin, a drug shown to boost mitochondrial biogenesis, largely mitigates the impact of *POLG* mutations, indicating therapeutic potential. Although metformin can inhibit mitochondrial complex I activity at low dosage, data from our previous study^[^ ^26^ ^]^ and other reports suggests that higher doses (250 – 1000 µm) of metformin increase complex I activity.^[^ ^55^ ^]^ We tested metformin and found that 250 µm for 2 months improved the mitochondrial deficit and partly mitigated the neuronal loss. These results nominate metformin as a potential candidate for clinical trials in POLG patients, subject to further preclinical validation.

Our findings must be interpreted in the light of the following limitation: we used healthy, age‐matched control iPSCs and patient‐specific iPSCs, which were differentiated into cortical organoids. We are fully aware that the current state‐of‐the‐art in iPSC research is the use of genetically corrected isogenic controls. Therefore, we generated isogenic cell lines for the homozygous WS5A patient. However, it has been reported that the high efficiency of genome cleavage and repair makes the introduction of heterozygous alleles by standard CRISPR/Cas9 technology difficult.^[^ ^30^ ^]^ Thus, we felt that the presence of compound heterozygous mutations, such as were present in the patient used in this study, made the generation of isogenic controls impractical. We feel, nevertheless, that our results are important and if we look only at the patient organoids, internally consistent. They show a clear abnormality and response to metformin that brings them in line with what is seen in controls.

Despite the advances represented by our POLG organoid model, there are limitations to be addressed. In our paradigm, the organoid culture method suppressed mesoderm‐derived immune cell/microglia presence^[^ ^56^, ^57^ ^]^ in order to achieve a more consistent forebrain identity and cell composition. This allowed us to address initial astroglia and neuron dependent pathologies without immune or secondary inflammatory responses. However, in future, the supplementation of POLG organoid models with microglia/allogeneic immune cells could allow us to address any immune‐related pathogenesis in POLG‐related disease. Evidence suggests that neuroinflammation and mitochondrial dysfunction may trigger a vicious circle: dysfunctional mitochondria induce inflammation, and inflammation induces mitochondrial dysfunction.^[^ ^58^ ^]^ It would be exciting, therefore, to address how immune cells affected by the *POLG* mutation contribute to cell vulnerability and disease pathogenesis in POLG organoids.

Our findings, based on comprehensive scRNA‐seq analysis, shed light on the diverse mechanisms through which metformin exerts its therapeutic effects in the context of POLG‐related diseases. A critical observation from our study is the ability of metformin to significantly alter the balance between neuronal and glial cell populations. The increase in neuronal cells and concurrent decrease in astrocytic cells following metformin treatment highlight its role in modulating neurogenesis and gliogenesis pathways. This suggests that metformin may contribute to mitigating reactive gliosis, a common response in neurodegenerative conditions associated with *POLG* mutations. The upregulation of mitochondrial markers such as TOMM20 and NDUFB10 in metformin‐treated organoids is particularly noteworthy. This enhancement in mitochondrial biogenesis and function is vital, given the mitochondrial dysfunctions characteristic of *POLG* mutations. Additionally, the elevation in TFAM levels, indicative of improved mtDNA stability, underscores metformin's role in maintaining mitochondrial integrity. Metformin treatment induced significant gene expression changes, with a downregulation of genes associated with neurodegenerative pathways and an upregulation of those involved in neuronal migration and neurotransmitter transport. This pattern reflects metformin's potential in promoting neuronal health and connectivity. Furthermore, the alteration in the MARK and MTOR signaling pathways suggests metformin's broader impact on cellular signaling, which is crucial for cell survival, growth, and metabolism. Notable changes were observed in cellular components, including the mitochondrial membrane and synaptic structures. These findings indicate metformin's profound impact on the structural and functional integrity of neurons, enhancing their resilience and functionality in the context of mitochondrial impairments due to *POLG* mutations.

Our study highlights the unparalleled benefits of using the 3D cerebral organoid model. This model excels in differentiating and producing a variety of brain cell types commonly found in the human brain, such as neurons, astrocytes, and neural stem cells. But it's not just about cellular differentiation: our in‐depth histological and functional analyses reveal that these 3D cerebral organoids closely mimic the human brain in both structure and functionality, closely resembling genuine brain tissue. What sets our study apart is our emphasis on the differentiation of POLG cerebral organoids. These specialized 3D constructs not only display molecular markers indicative of POLG‐related diseases but also shed light on the distinct physiological and functional characteristics of these conditions. This provides an unparalleled resource for studying POLG‐linked disorders, enabling research to encompass interactions among a variety of cell types, rather than just focusing on individual cells. By harnessing the potential of these 3D cerebral organoids, we are well‐positioned to explore the complex relationships between different brain cells, uncover the molecular underpinnings of diseases, and lead the charge in drug testing and validation. Most importantly, our study provides significant insights into the complex action of metformin in POLG‐related diseases. The drug appears to exert a multifaceted influence, encompassing not only the amelioration of mitochondrial dysfunction but also beneficial alterations in cell population dynamics, gene expression, and key cellular signaling pathways. These observations offer a deeper understanding of metformin's therapeutic potential and lay the groundwork for future research into targeted treatments for mitochondrial disorders.

## 4 Experimental Section

### Ethics Approval

The project was approved by the Western Norway Committee for Ethics in Health Research (REK nr. 2012/919).

### Generation and Maintenance of iPSCs

Skin fibroblasts were obtained from punch biopsies of two patients carrying *POLG* mutations. One patient was homozygous for the c.2243G > C; p.W748S mutation (WS5A), and the other was compound heterozygous for the c.1399G > A/c.2243G > C; p.A467T/W748S mutations (CP2A). The patient identifiers WS5A and CP2A were previously used in the published work.^[^ ^12^, ^13^, ^25^, ^26^ ^]^ Isogenic control for homozygous for the c.2243G > C; p.W748S mutation (WS5A), along with controls who were matched for age and gender, were included in the study. This group also encompassed iPSCs derived from Detroit 551 fibroblasts (ATCC CCL 110TM), CCD‐1079Sk fibroblasts (ATCC CRL‐2097), and AG05836. To generate iPSCs, the skin fibroblasts were reprogrammed using retroviral or Sendai virus vectors containing the coding sequences of human OCT4, SOX2, KLF4, and c‐MYC. The iPSCs were maintained under feeder‐free conditions as previously described.^[^ ^12^, ^13^, ^27^, ^59^ ^]^ All iPSC lines, including the patient‐derived lines and control lines, were maintained following established protocols.^[^ ^12^, ^13^, ^25^, ^26^ ^]^ Regular monitoring for mycoplasma contamination was performed using the Myco Alert Mycoplasma Detection Kit (Lonza, #LT07‐218) to ensure the integrity of the cell lines.

### CRISPR‐Cas9 Mediated Cell Line Generation

CRISPR‐Cas9 technology was employed to generate isogenic POLG W748S iPSCs, with the procedure conducted by Synthego Corporation, Redwood City, CA, USA. The process involved the use of ribonucleoprotein complexes, comprising Cas9 protein and synthetically modified sgRNA developed by Synthego. These complexes were introduced into the cells via electroporation, along with a single‐stranded oligodeoxynucleotide (ssODN) acting as a donor template, following Synthego's proprietary protocol.

Post‐electroporation, the editing efficiency was evaluated after a 48 h recovery period. A sample of the cells was taken for genomic DNA extraction, followed by PCR amplification and Sanger sequencing of the targeted region. The resulting sequence data was analyzed using Synthego's Inference of CRISPR edits (ICE) software, available at ice.synthego.com, to assess the editing outcomes.

For the establishment of monoclonal cell lines, cells from the edited pool were distributed at a density of one cell per well, using a single‐cell printer into either 96‐well or 384‐well plates. Regular imaging, conducted every three days, ensured that each clonal population originated from a single cell. These clonal populations were then subjected to the PCR‐Sanger‐ICE genotyping strategy for screening and identification, ensuring precise characterization of the genetic alterations.

### Karyotype Analyses

Human G banding karyotyping was performed using the protocol as reported previously.

### Generation of Cortical Organoid

To generate cortical organoids from iPSCs, a previously described protocol was followed.^[^ ^60^, ^61^ ^]^ Initially, feeder‐free iPSCs were cultured in E8 medium for a minimum of 7 days prior to differentiation. The iPSC colonies were dissociated using Accutase (Life Technologies, #A11105‐01) in a 1:1 mixture with phosphate‐buffered saline (PBS), incubated at 37 °C for 10 min, and then centrifuged at 100 × g for 3 min. The resulting single cells were diluted in neural induction media (Table S1, Supporting Information) and ≈9000 viable cells were seeded into each well of 96‐well ultra‐low attachment tissue culture plates (S‐BIO, #MS‐9096UZ) in 150 µl of neural induction media. The plates were kept in suspension and rotated at 85 rpm for 24 h, with the addition of 50 µm Y‐27632 Rock Inhibitor (Tocris Bioscience, #1254) to promote the formation of EBs. To minimize undirected differentiation, dual SMAD inhibition and canonical Wnt inhibition were employed during the initial phase. On day 2, half of the media in each well was replaced with human neural induction media containing 50 µm ROCK inhibitor. On days 4, 6, and 8, 100 µl of the medium was replaced with 150 µl of neural induction media without the ROCK inhibitor. After 10 days, the EBs were transferred to 6‐well ultra‐low attachment tissue culture plates (Corning, #3471) and cultured in neural differentiation media (Table S2, Supporting Information) without vitamin A for the next 8 days using an orbital shaker. This step aimed to induce the formation of cortical organoids. On day 18, the organoids were further matured in neural differentiation media supplemented with vitamin A (Table S3, Supporting Information). Media changes were performed every 3–4 days, and the addition of Brain Derived Neurotrophic Factor (BDNF, PeproTech, #450‐02) and ascorbic acid (Sigma‐Aldrich, #92902‐500G) facilitated long‐term neural maturation.

### Metformin Treatment of Cortical Organoids

For phenotype rescue experiments, organoids were treated with 250 µm metformin (Sigma‐Aldrich, #317 240) from day 6 of differentiation. The medium was changed every two days, and the organoids were grown for 3 months. An equivalent amount of vehicle of Dimethylsulfoxide (DMSO, Sigma‐Aldrich, #153 087) was added to grow untreated organoids.

### Snap Freezing and Embedding

Each human cerebral organoid was fixed in 4% paraformaldehyde (PFA, Thermo Fisher Scientific, #28 908) in PBS overnight at 4 °C, dehydrated with 30% sucrose in PBS. The samples were then embedded in gelatin solution (Sigma‐Aldrich, #G1393) and snap frozen in boiled liquid nitrogen. The samples were then embedded in optimal cutting temperature (O.C.T) compound (Thermo Fisher Scientific, #23‐730‐57). Cryostat sections (15 µm) were cut and mounted Superfrost adhesion slide (Thermo Fisher Scientific, #J1800AMNZ).

### Immunofluorescence Staining

Mounted sections were incubated for 1 h at room temperature with and blocked using blocking buffer containing (Sigma‐Aldrich, #G9023) and 0.1% (v/v) Triton X100 (Sigma‐Aldrich, #9036‐19‐5), and then incubated with primary antibodies diluted in blocking solution overnight at 4 °C. The following primary antibodies were used for immunostaining: Nanog (rabbit, 1:100; Abcam, #ab80892), OCT4 (rabbit, 1:100; Abcam, #ab19857), SSEA4 (mouse, 1:200; Abcam, #ab16287,), NeuN (rabbit, 1:500; Cell Signaling, #24307S), MAP2 (rabbit, 1:1000; Abcam, #ab5392), β‐tubulin III (TUJ1) (mouse, 1:1000; Abcam, #ab78078), Synaptophysin (mouse, 1:500; Proteintech, #17785‐1‐AP), PSD‐95 (rabbit, 1:500; Proteintech, #20665‐1‐AP), SOX2 (rabbit, 1:100; Abcam, #ab97959), NDUFB10 (rabbit, 1:350; Abcam 196 019), TFAM (mouse, 1:1000; Abcam #ab119684), TOMM20 (mouse, 1:350; Abcam, #ab56783), GFAP (chicken, 1:500; Abcam #ab4674), DCX (mouse, 1:200; Abcam #ab135349), SATB2 (rabbit, 1:400; Abcam #ab4674), CTIP2 (rat, 1:500; Abcam, #ab18465), OLIG2 (rabbit, 1:500; Abcam, #ab42453). Alexa Fluor Dyes (Invitrogen, #A11008, #A21449, #A11012, #A21141, #A11042, #A21236) were used at 1:800 dilution as secondary antibodies. Slides were mounted using ProLong diamond antifade mounting medium (SouthernBiotech, #0100‐20), and analyzed using the Leica TCS SP8 STED 3X (Leica microsystems).

### Fluorescence Imaging Analysis

Immunofluorescence images were quantitatively measured using Image J software (Image J 1.52a; Wayne Rasband National Institutes of Health, USA). Six to ten regions within cortical layers were randomly selected for quantitative evaluation. To measure fluorescence intensity, single‐channel images were converted to 8‐bit images using Image J. To eliminate errors caused by manually selecting thresholds for different photos, default thresholds were used. Next, the default algorithm, and parameters were choosed. Finally, the fluorescence intensity was measured by the mean gray value (Mean) with the following formula: Mean = Integrated Density (IntDen)/Area. Data were analyzed and plotted with GraphPad Prism 8.0.2 software (GraphPad Software, Inc). Details of statistical tests and p‐values are described in Table S5 (Supporting Information).

### ScRNA‐seq) and Data Analysis

*Organoid Dissociation and Single Cell Isolation*: To collect the organoids, they were removed from the culture medium and washed with 1x PBS (Invitrogen, #10010‐23). The organoids were then finely cut into pieces of 1–2 mm using ophthalmic scissors. Next, the organoid pieces were digested in 2 ml of CellLive Tissue Dissociation Solution (Singleron Biotechnologies, #1 190 062) at 37 °C for 15 min in a 15‐ml centrifuge tube (Sarstedt, #62.5544.003), with continuous agitation on a thermal shaker. The degree of dissociation was periodically checked under a light microscope. Following digestion, the suspension was filtered through a 40‐µm sterile strainer (Greiner, #542 040). The cells were then centrifuged at 350 x g for 5 min at 4 °C, and the resulting cell pellets were resuspended in 1 ml of PBS. To assess cell viability and count, the cells were stained with a 0.4% w/v solution of Trypan Blue (Gibco, #15250‐061). The cell number and viability were determined using a hemocytometer under a light microscope.

*ScRNA‐seq Library Preparation*: scRNA‐seq libraries were prepared using the GEXSCOPE Single Cell RNAseq Library Kit (Singleron Biotechnologies, #4 161 031) following the manufacturer's instructions. Briefly, the single‐cell suspension was adjusted to a concentration of 3 × 10^5^ cells ml with PBS and loaded onto a microfluidic chip to capture 6000 cells. Paramagnetic beads conjugated to oligo(dT) probes with unique molecular identifiers (UMIs) and barcodes were added, and the cells were lysed. The polyadenylated mRNA bound to the beads was extracted, reverse transcribed into cDNA, and amplified by PCR. The resulting cDNA was fragmented and ligated to indexed Illumina adapters. The final amplified library's fragment size distribution was analyzed using an Agilent Fragment Analyzer.

*Library Sequencing*: The library concentration was calculated using the Qubit 4.0 fluorometer and the libraries were pooled in an equimolar fashion. The single cell libraries were sequenced on an Illumina NovaSeq 6000 using a 2 × 150‐bp approach to a final depth of 90 GB per library. The reads were demultiplexed according to the multiplexing index sequencing on Illumina's Base Cloud platform.

*Transcriptome Data Pre‐Processing*: The scRNA‐seq data was pre‐processed using the CeleScope software (v.1.3.0; www.github.com/singleron‐RD/CeleScope; Singleron Biotechnologies GmbH) to generate raw data with default parameters. Low quality reads were discarded, and the remaining sequences were mapped to the human reference genome GRCh38 using STAR (https://github.com/alexdobin/STAR). Gene annotation was performed using Ensembl 92. The assignment of reads to genes was done using featureCount (https://subread.sourceforge.net/), resulting in a count matrix file that contained the number of Unique Molecular Identifiers (UMIs) for each gene within each cell.

Subsequent analysis was carried out using the scanpy package in Python. Quality control metrics, such as the number of genes detected per cell (nFeature_RNA) and the percentage of mitochondrial UMIs (percent_mt), were extracted from the gene count matrix using the calculate_qc_metrics function. To remove non‐viable cells, cells with a high percentage of mitochondrial counts (>20%) were filtered out. Cells with a high number of detected genes (>5000), indicative of potential doublets, were also excluded. Additionally, cell debris characterized by a low number of detected genes (<200) were removed from the dataset.

### Sample Integration, Dimensionality Reduction, and Cell Clustering

The downstream bioinformatic analysis was performed by combining three samples using the *concatenate* function from the anndata package in Python. Data were log‐normalized such that the total count for each cell was 10000. Highly variable genes (HVGs) were identified using dispersion‐based methods with mean between 0.1 and 8 and dispersion of 0.5 and above using the function highly_variable_genes from scanpy. Principal component analysis (PCA) was performed on expressions from HVGs. The top 17 principal components were retrained from 50 by checking explained variance ratio of PCA instances. Neighbors’ function was used in *scanpy* to calculate the neighborhood graph with 20 neighbors and 17 PC. The Uniform Manifold Approximation and Projection (UMAP) algorithm was used to calculate the reduced dimensions (Figure S1, Supporting Information) from neighborhood graph with minimum distance of 0.5, spread scale of 1 and 200 maximum iterations. The cell clusters were identified using the unsupervised network‐based leiden algorithm^[^ ^62^ ^]^ provided in the scanpy package with a resolution of 0.5 on the top 17 principal components.

### Cell Type Annotation

To identify distinct cell populations based on shared and unique patterns of gene expression, dimensionality reduction and unsupervised cell clustering were performed using single cell multi‐resolution marker‐based annotation scMRMA^[^ ^63^ ^]^ (https://github.com/JiaLiVUMC/scMRMA) with cutoff of significant p value smaller than 0.05 for the fisher test for the significant cell type enrichment from 20 nearest neighbors. Cell type marker genes were obtained from the PanglaoDB database (https://panglaodb.se/).

### DEG Analysis

The marker genes of the individual clusters were found by using the differential gene expression analysis algorithm scanpy.tl.rank_genes_groups in Python with the following parameters sc.tl.rank_genes_groups (adata, groupby = ’leiden’, method = “wilcoxon”, corr_method = “bonferroni”, use_raw = False). A p‐value of 0.05 was used to keep the DEGs that were significant. The top 9 DEGs were shown for each cluster in the figures.

### Gene Enrichment Analysis

Gene enrichment analysis was performed with the package *gseapy* in Python with the databases “GO_Biological_Process_2021”, “GO_Molecular_Function_2021”, “GO_Cellular_Component_2021”, “Reactome_2016”, “KEGG_2016” and “KEA_2015”. The information on the software used for scRNA‐seq analysis was listed in Table S4 (Supporting Information).

### Statistical Analysis

The data were presented as mean ± standard deviation (SD) for samples with a minimum size of three. The normality of the data distribution was assessed using the Shapiro‐Wilk test. Outliers were identified using the ROUT method. For variables with a non‐normal distribution, statistical significance was determined using the Mann‐Whitney U‐test, while variables with a normal distribution were analyzed using a two‐sided Student's t‐test. Graphs and statistical analyses were performed using GraphPad Prism 8.0.2 software (GraphPad Software, Inc). A p‐value of ≤ 0.05 was considered statistically significant. Detailed information on the specific statistical tests conducted and corresponding p‐values can be found in Table S5 (Supporting Information).

## Conflict of Interest

The authors declare no conflict of interest.

## Author Contributions

K.L. and A.C. conceptualized the study. K.L., A.C., and Y.H. were involved in methodology. K.L., A.C., Y.H., T.Y., B.C.L. conducted the investigation. K.L. and A.C. wrote the original draft. K.L., A.C., Y.H., T.Y., B.C.L., G.J.S., C.T., and L.A.B. contributed to writing, review, and editing. K.L., L.A.B., and G.J.S. acquired funding. K.L. and L.A.B. provided resources. K.L. supervised the study. All authors have agreed to authorship.

## Supporting information

Supporting Information

## Data Availability

The data that support the findings of this study are available in the supplementary material of this article.
