# Supplementary material for: Hallmark Molecular and Pathological Features of POLG Disease are Recapitulated in Cerebral Organoids
Source: Adv Sci (Weinh). 2024 Mar 6;11(18):2307136. doi: 10.1002/advs.202307136 (PMC11095234; doi:10.1002/advs.202307136)
Supplement: Supplementary file 1 — Supporting Information [file ADVS-11-2307136-s001.pdf]

ADVANCED SCIENCE
Open Access

Supporting Information

for *Adv. Sci.*, DOI 10.1002/advs.202307136

Hallmark Molecular and Pathological Features of POLG Disease are Recapitulated in Cerebral Organoids

*Anbin Chen, Tsering Yangzom, Yu Hong, Bjørn Christian Lundberg, Gareth John Sullivan, Charalampos Tzoulis, Laurence A. Bindoff and Kristina Xiao Liang**

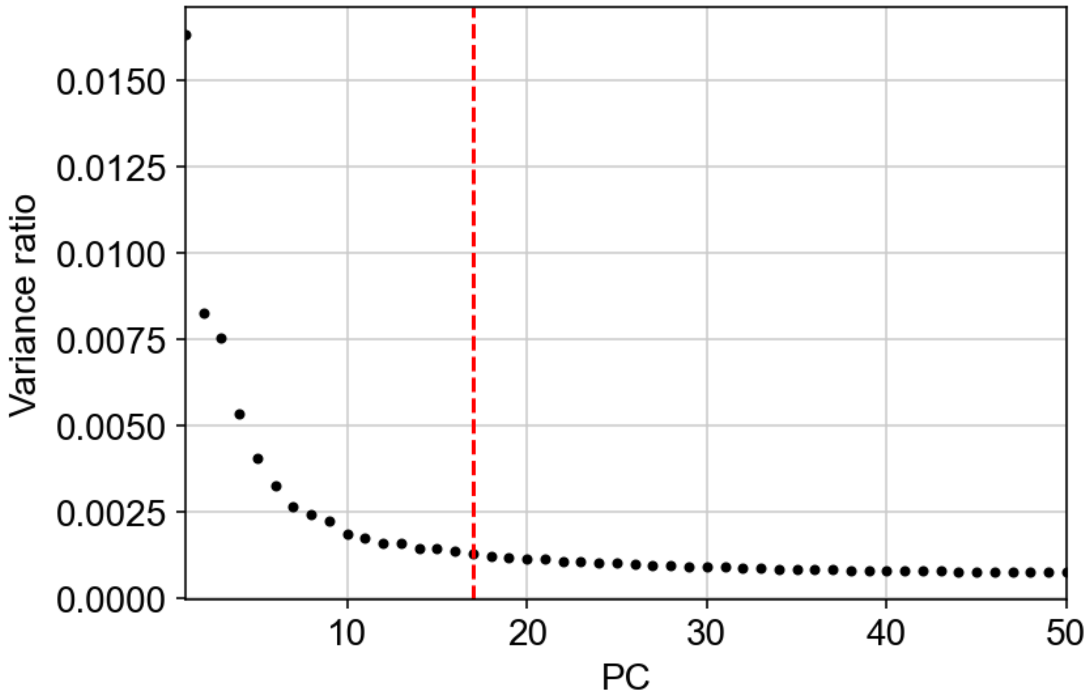

Supplementary Figure 1: Detection of number of principal components before reaching the plateau. The plateau is reached at the 17th principal component (shown with a dashed red line).

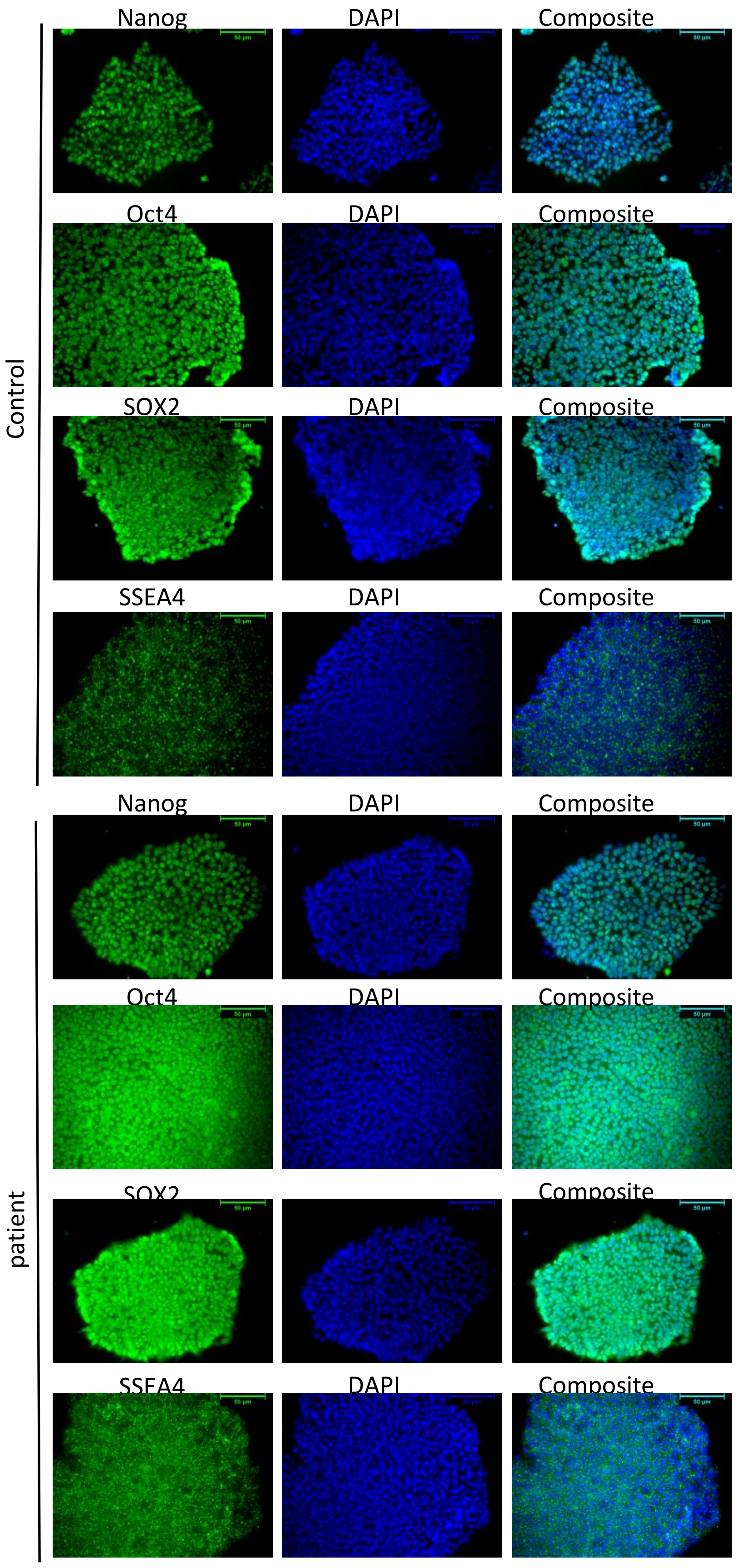

Supplementary Figure 2: Panel of pluripotency marker expression in control and patient iPSCs. *Scale bar is 50 µm.*

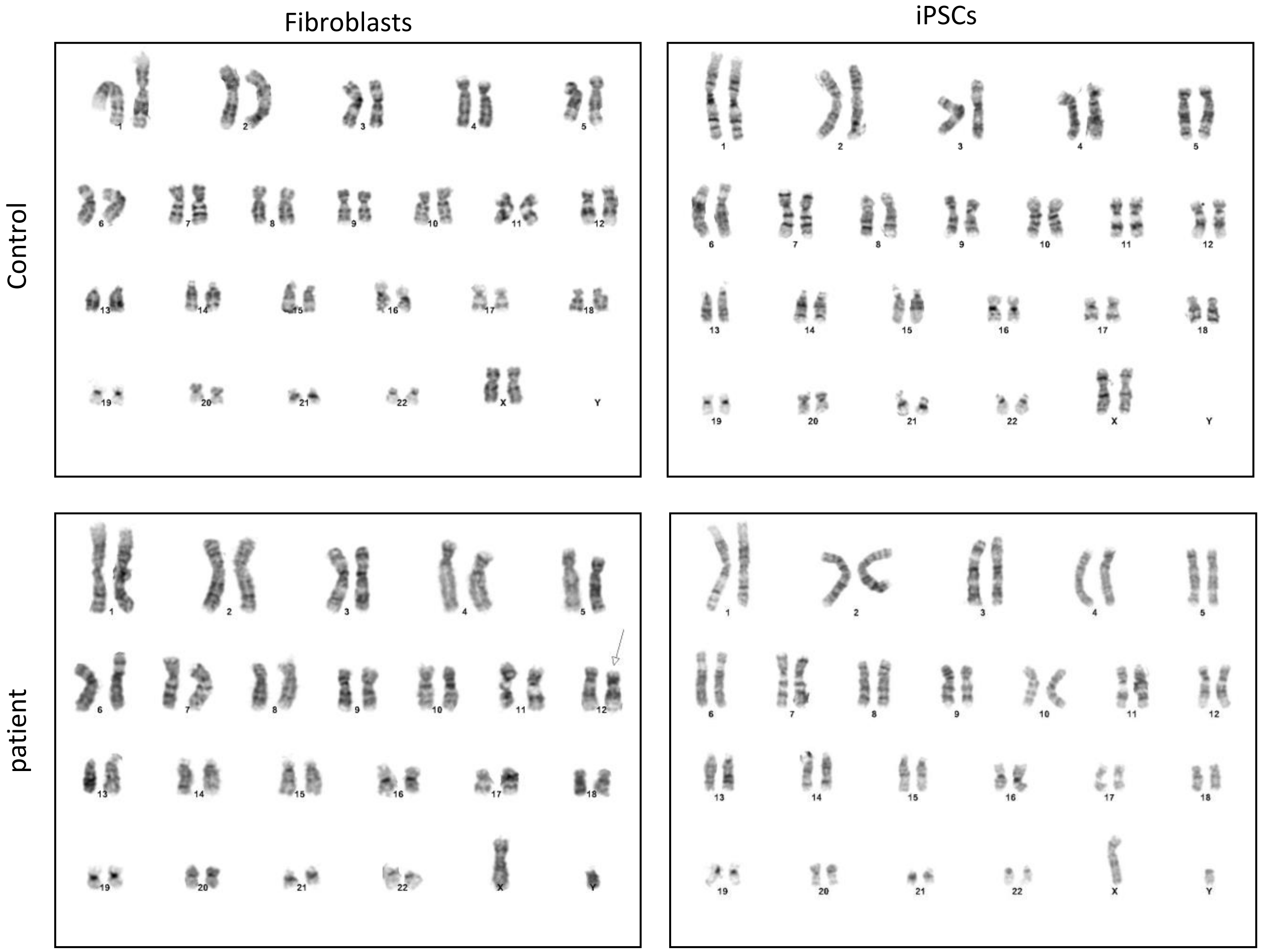

Supplementary Figure 3: Representative karyotypes for control and patient POLG fibroblasts and reprogrammed iPSC lines.

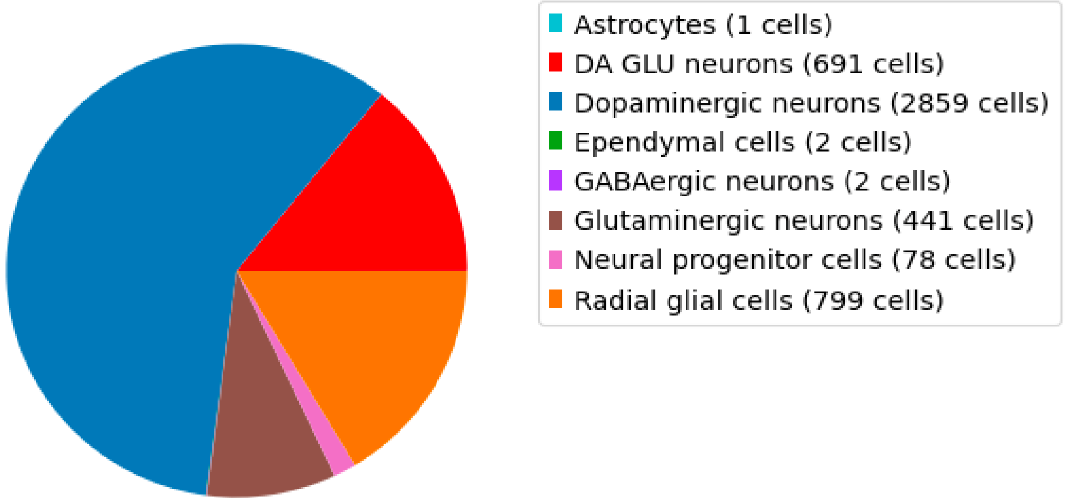

Supplementary Figure 4. Number of each cell cluster in scRNA-seq of control cortical organoids.

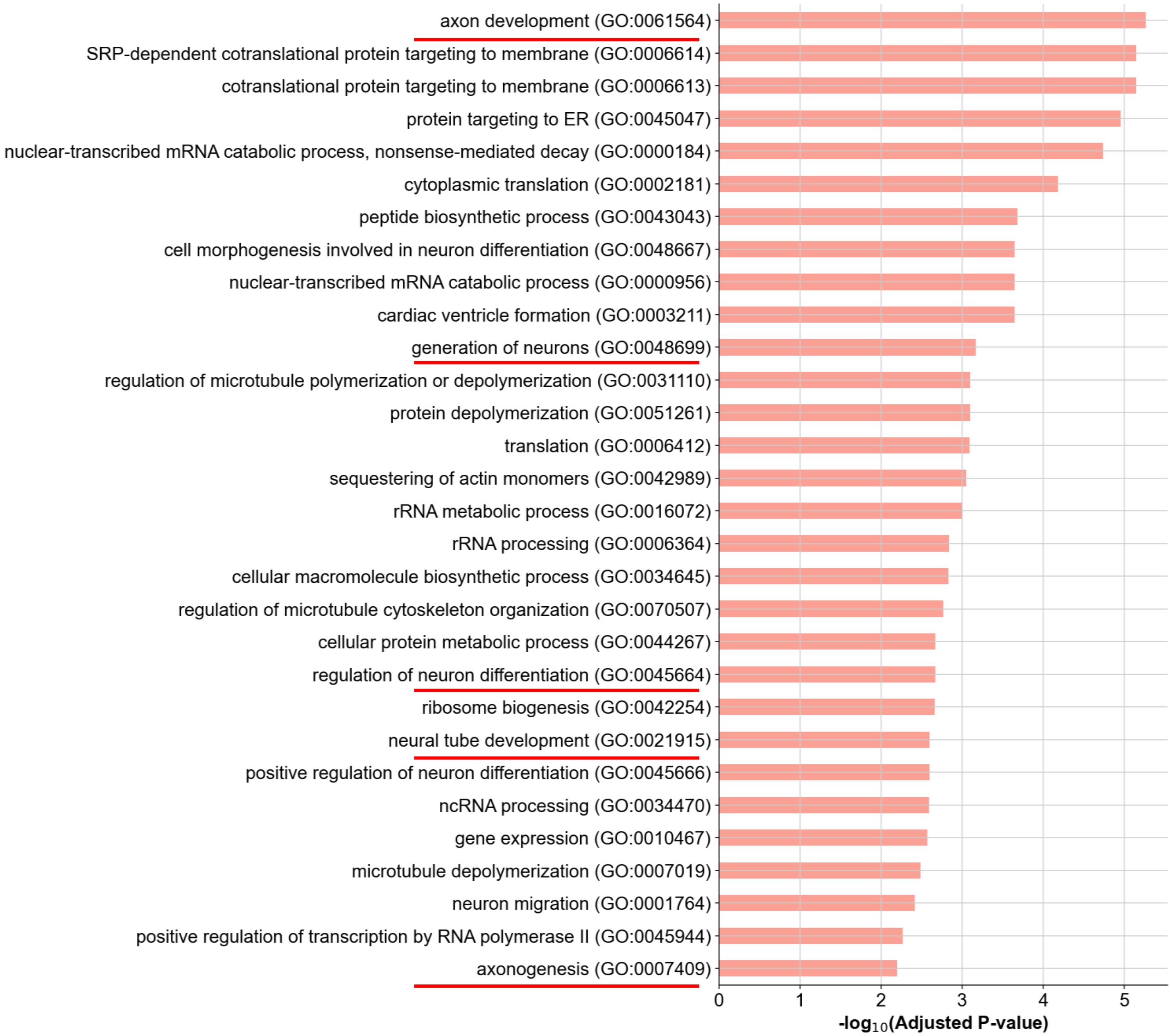

Supplementary Figure 5: GO terms in neuron population enriched in the analysis of control cortical organoids.

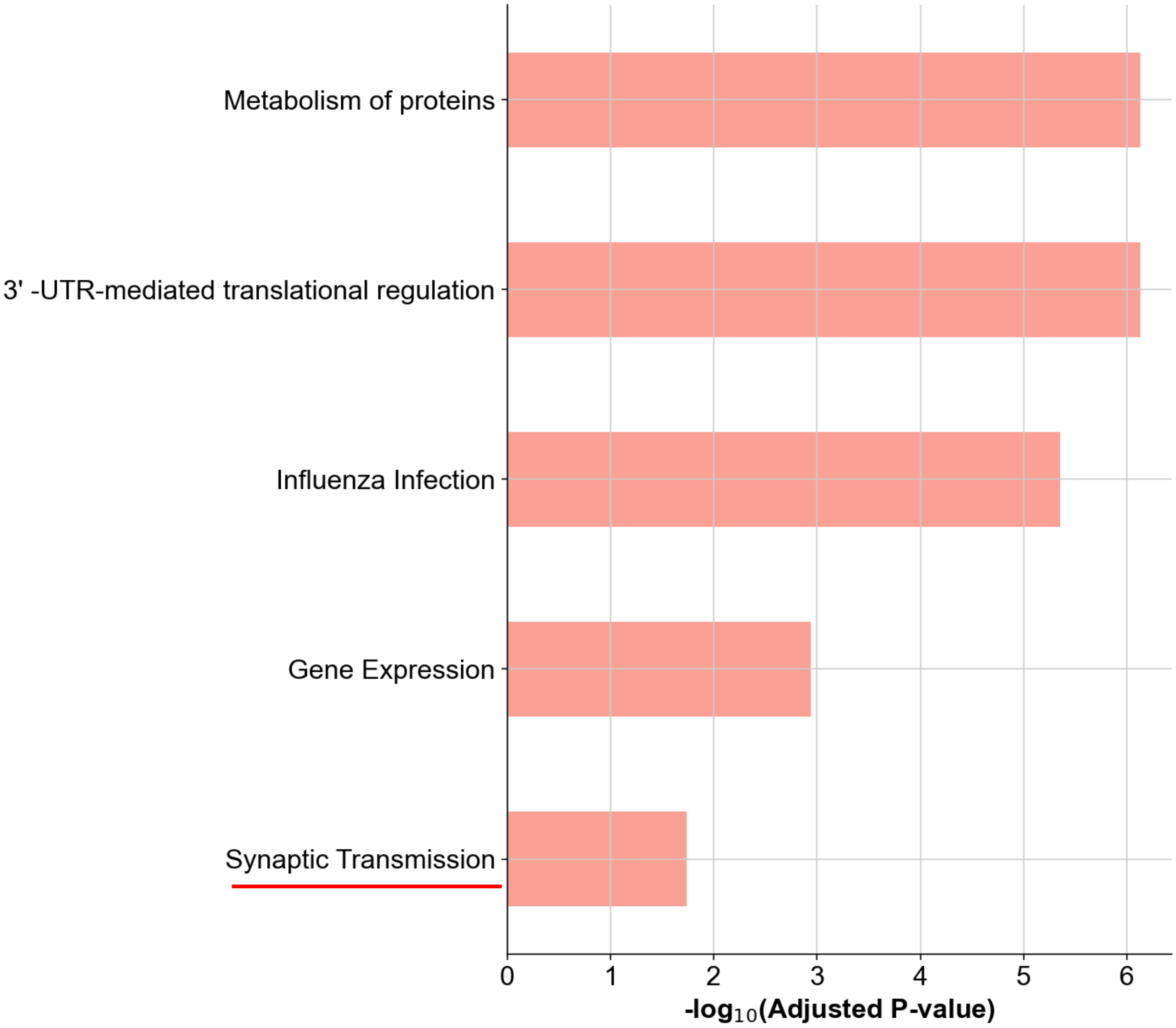

Supplementary Figure 6: Reactome terms enriched in the analysis of neuron population in control cortical organoids.

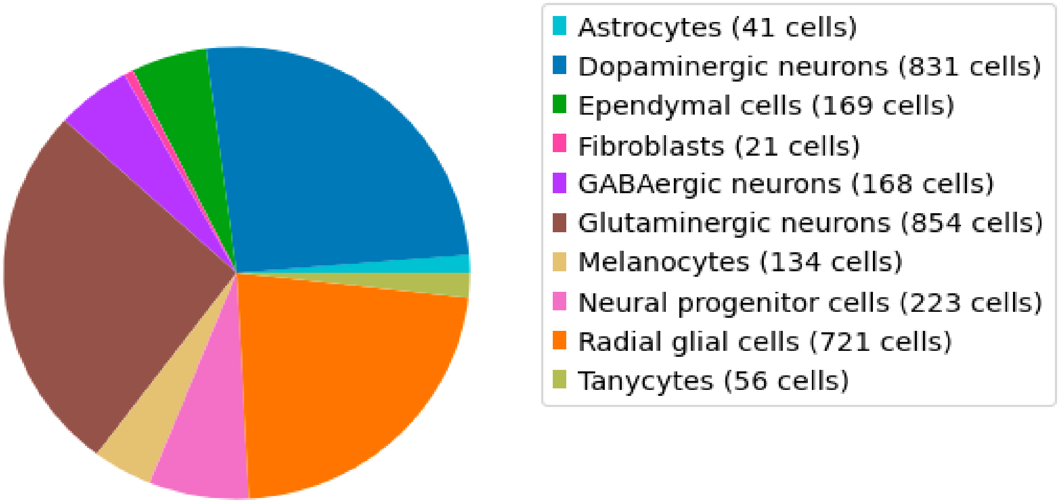

Supplementary Figure 7. Number of each cell cluster in scRNA-seq of patient cortical organoids.

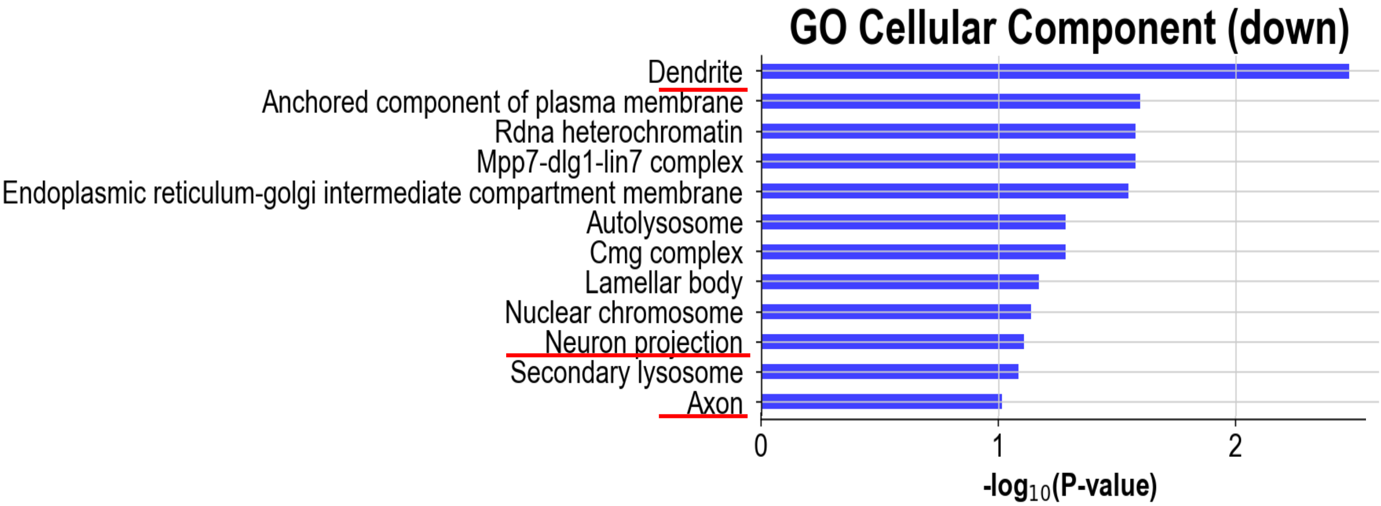

Supplementary Figure 8: GO cellular components enriched in the analysis of the downregulated DEGs of the neuron population in patient cortical organoids versus control.

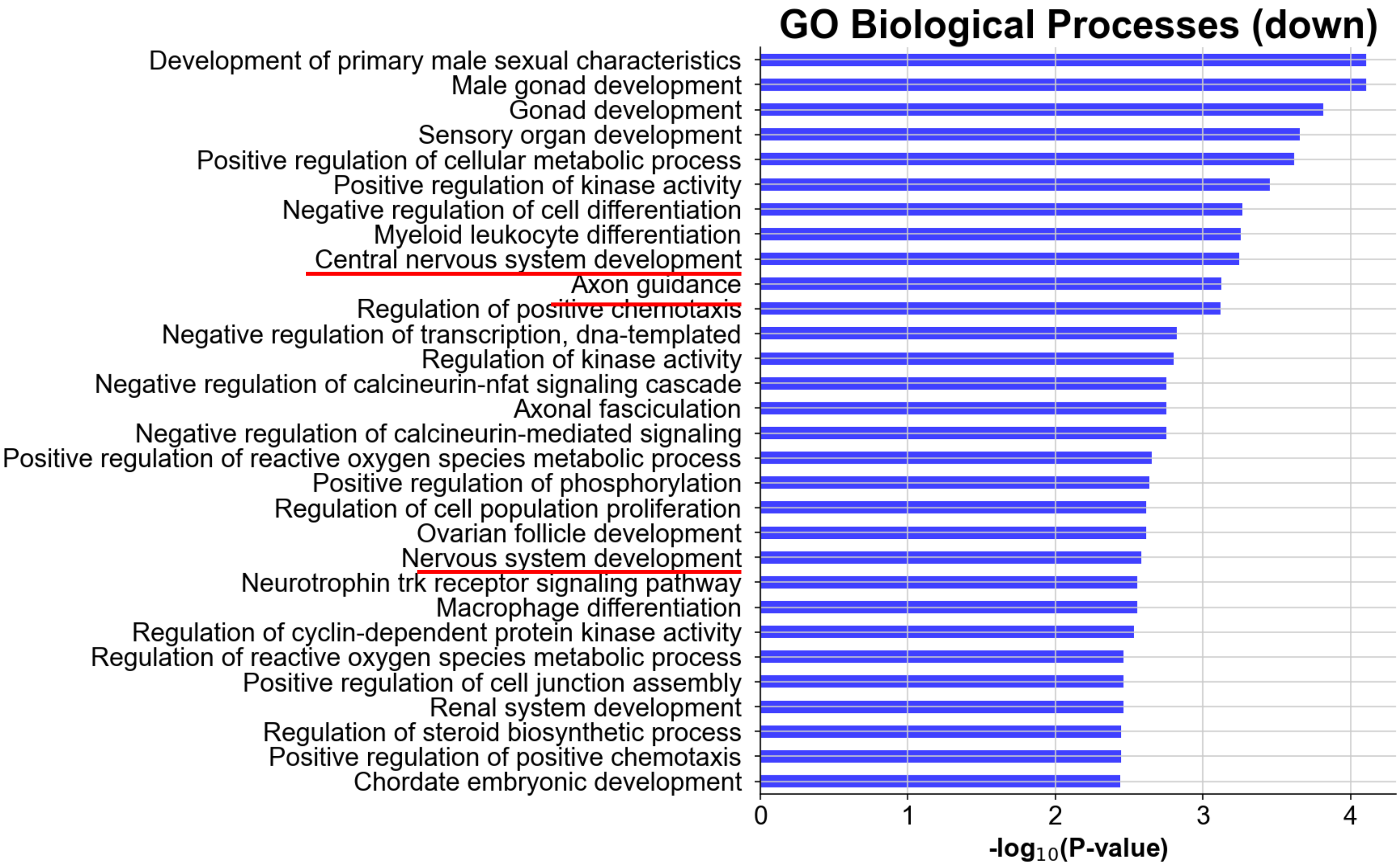

Supplementary Figure 9: GO biological processes enriched in the analysis of the downregulated DEGs of the glial population in patient cortical organoids versus control.

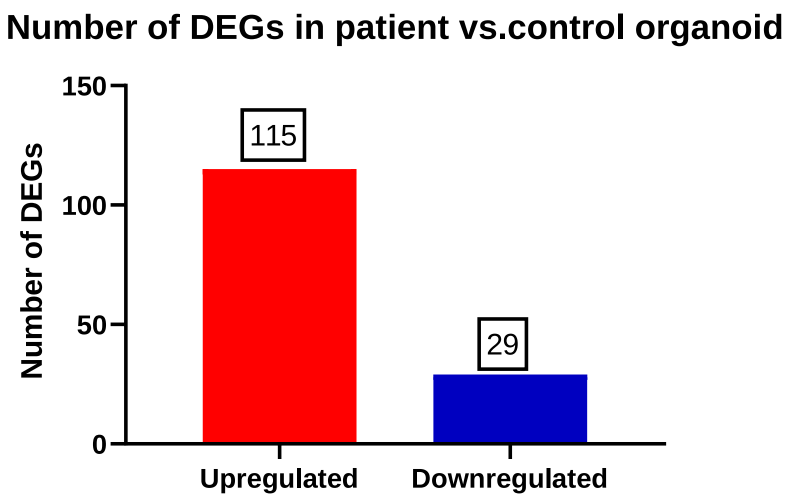

Supplementary Figure 10: Numbers of up- and downregulated DEGs pooled for the dopaminergic neuron population in patient cortical organoids versus control organoids.

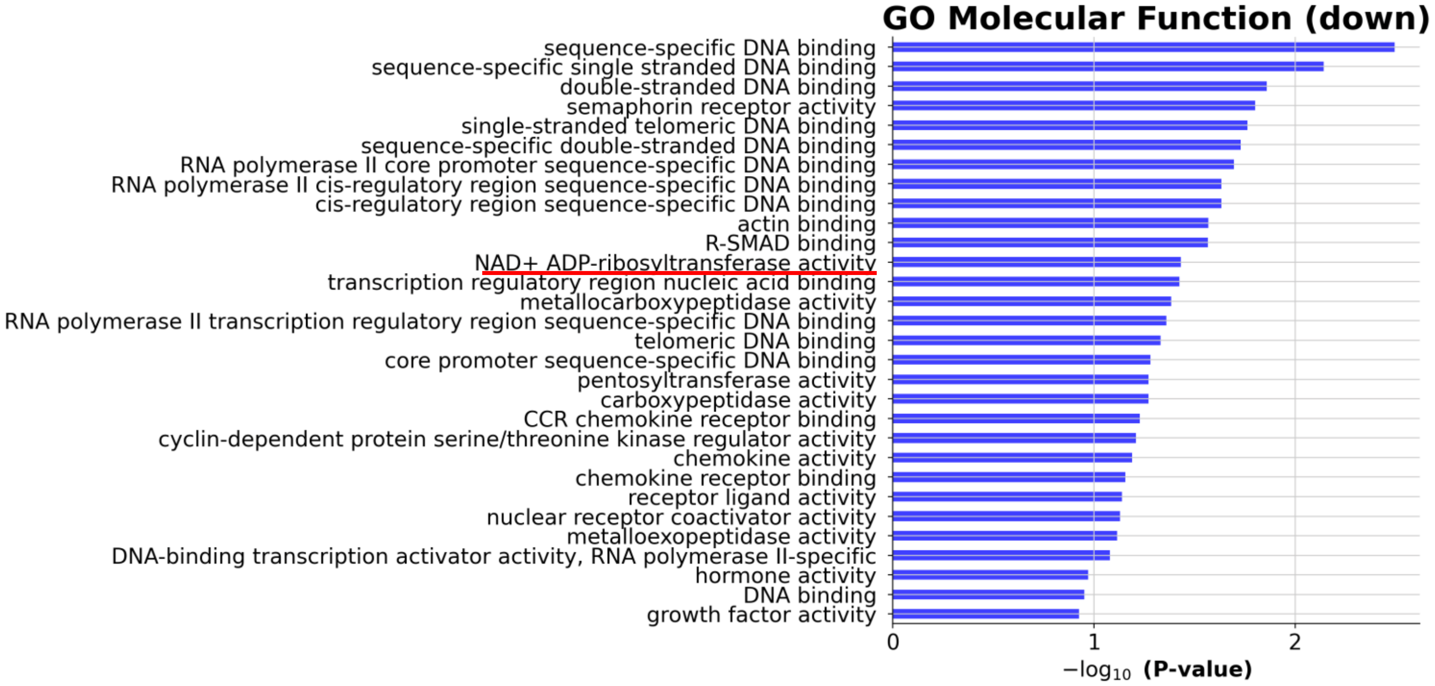

Supplementary Figure 11: GO molecular function processes enriched in the analysis of the downregulated DEGs of the dopaminergic neuron population in patient cortical organoids versus control.

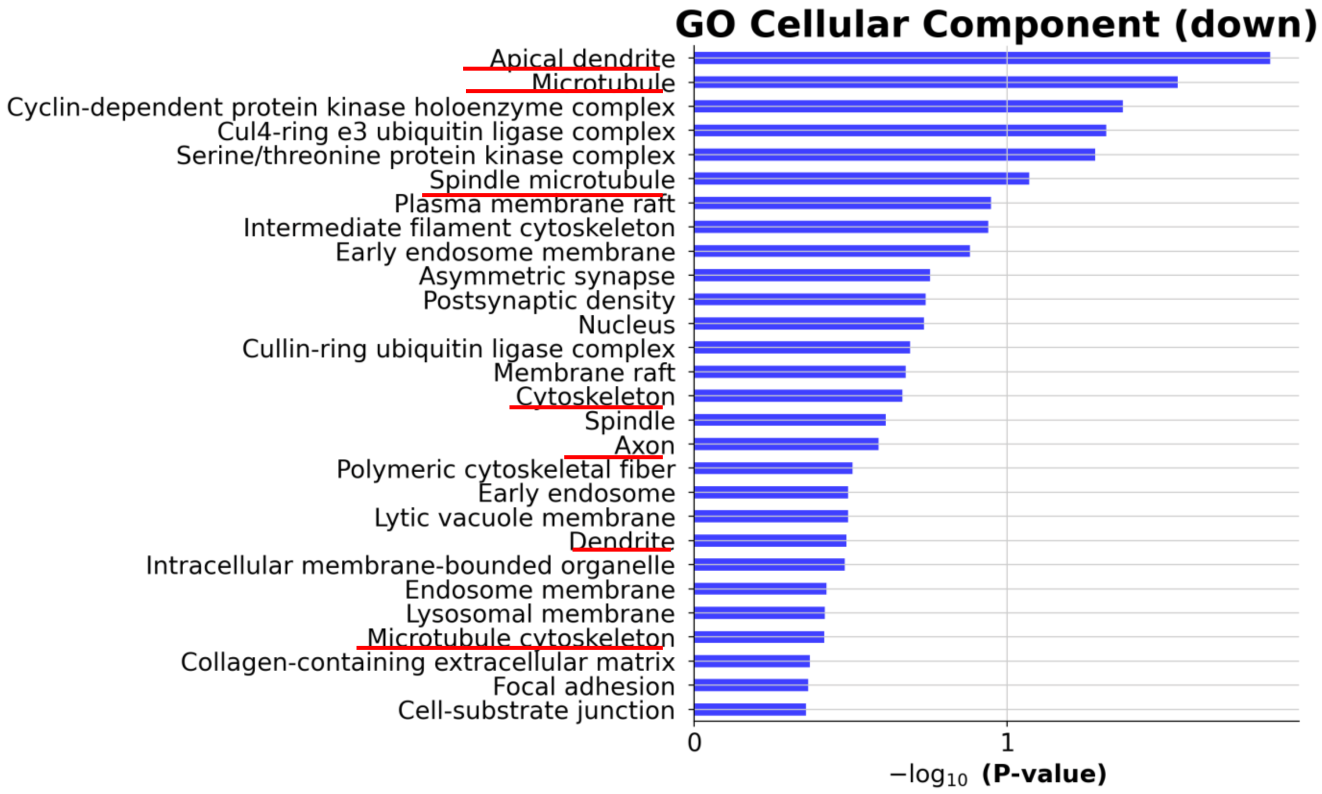

Supplementary Figure 12: GO cellular components enriched in the analysis of downregulated DEGs of the dopaminergic neuron population in patient cortical organoids versus control.

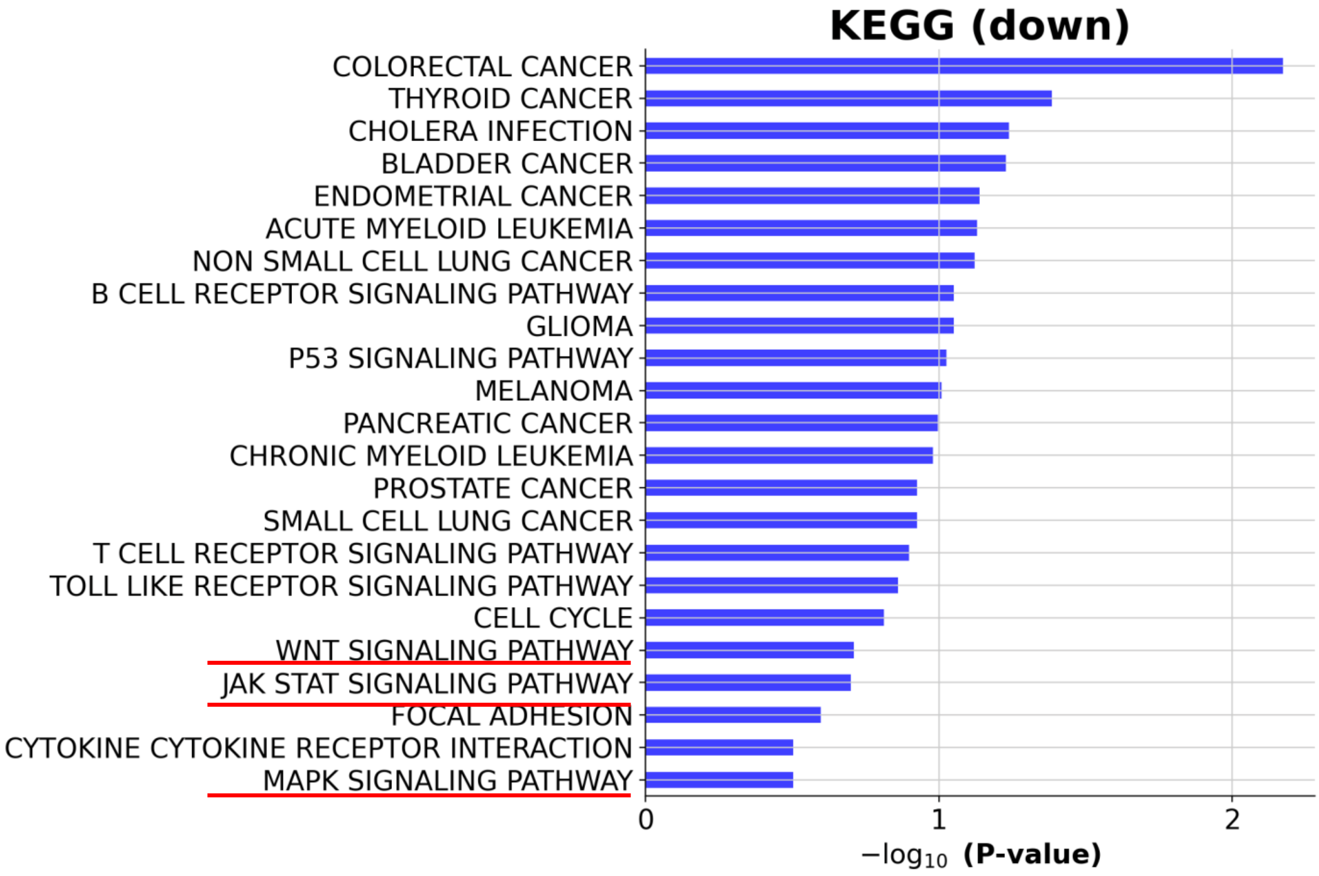

Supplementary Figure 13: KEGG pathways enriched in the analysis of downregulated DEGs of the dopaminergic neuron population in patient cortical organoids versus control.

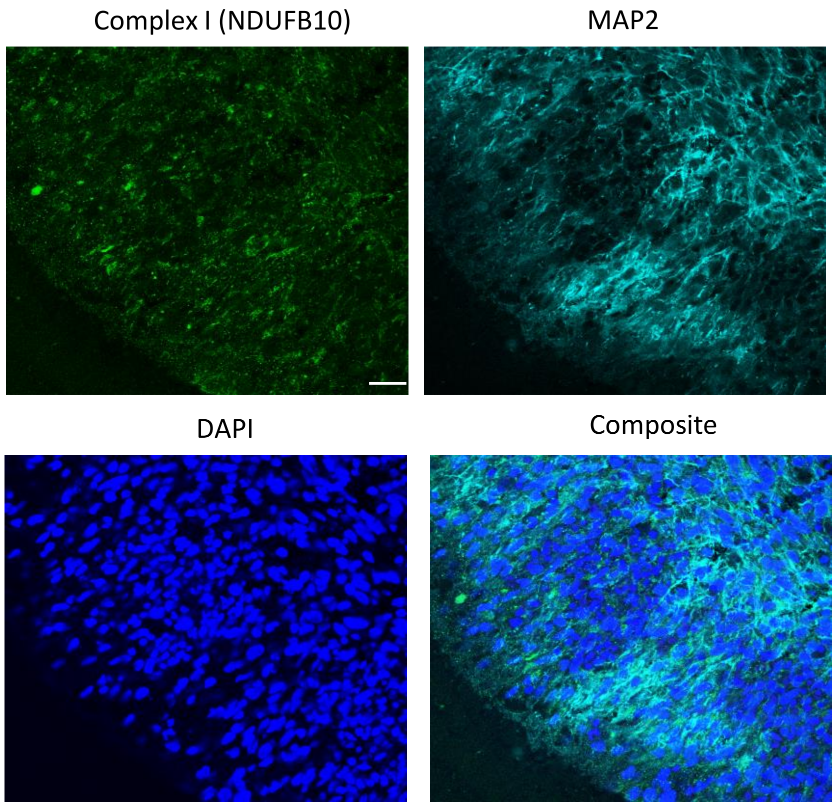

Supplemental Figure 14. Immunofluorescent imaging of cryo-sectioned organoids at 90 days, showing the staining of NDUFB10 (green), GAD65 (red), and MAP2 (purple) in patient organoids and patient organoids treated with metformin. Nuclei are stained with DAPI (blue). Scale bar is 50 µm.

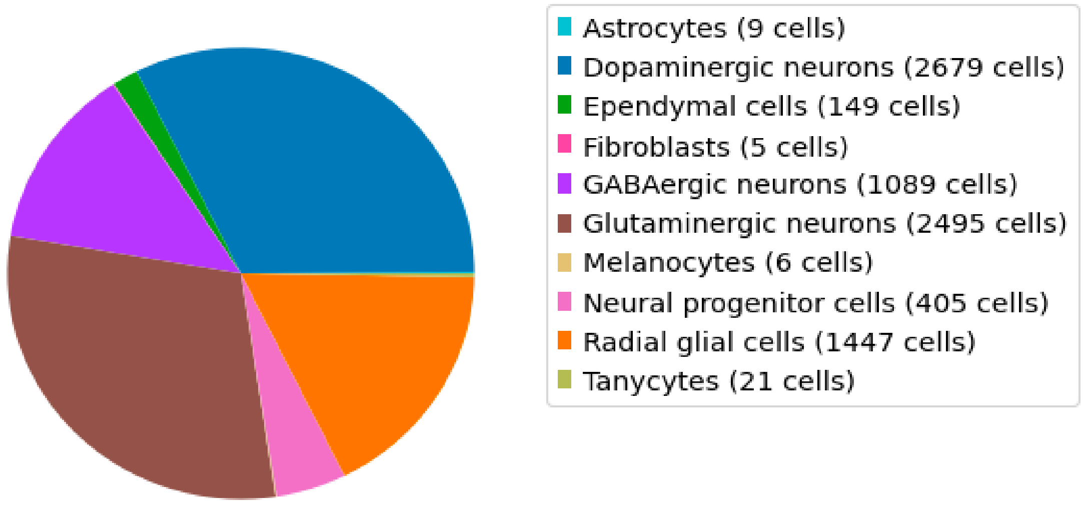

Supplementary Figure 15. Number of each cell cluster in scRNA-seq of patient cortical organoids treated with metformin.

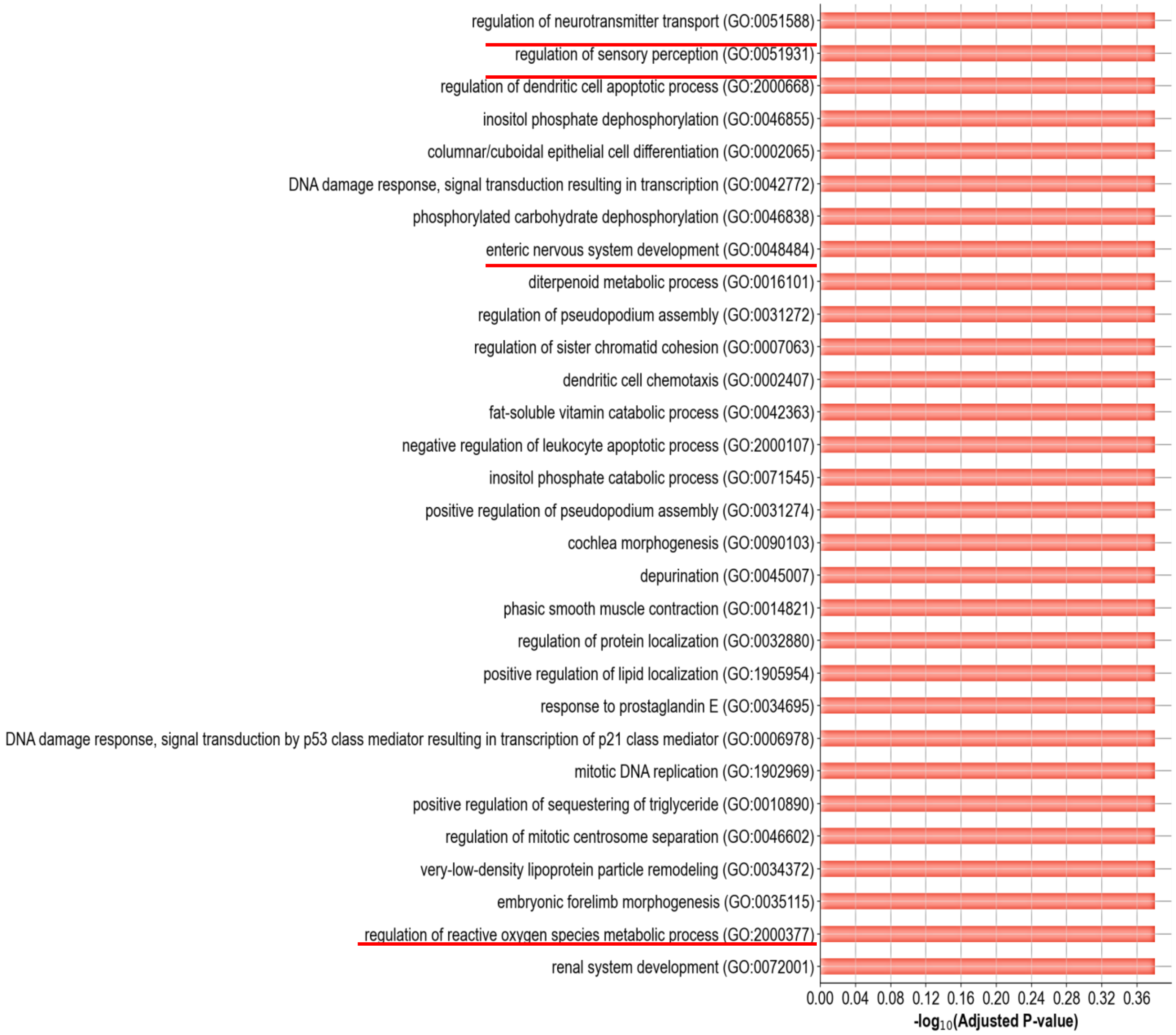

Supplementary Figure 16: GO terms enriched in the analysis of the upregulated DEGs of the neuron population in patient cortical organoids with metformin treatment versus untreated organoids.

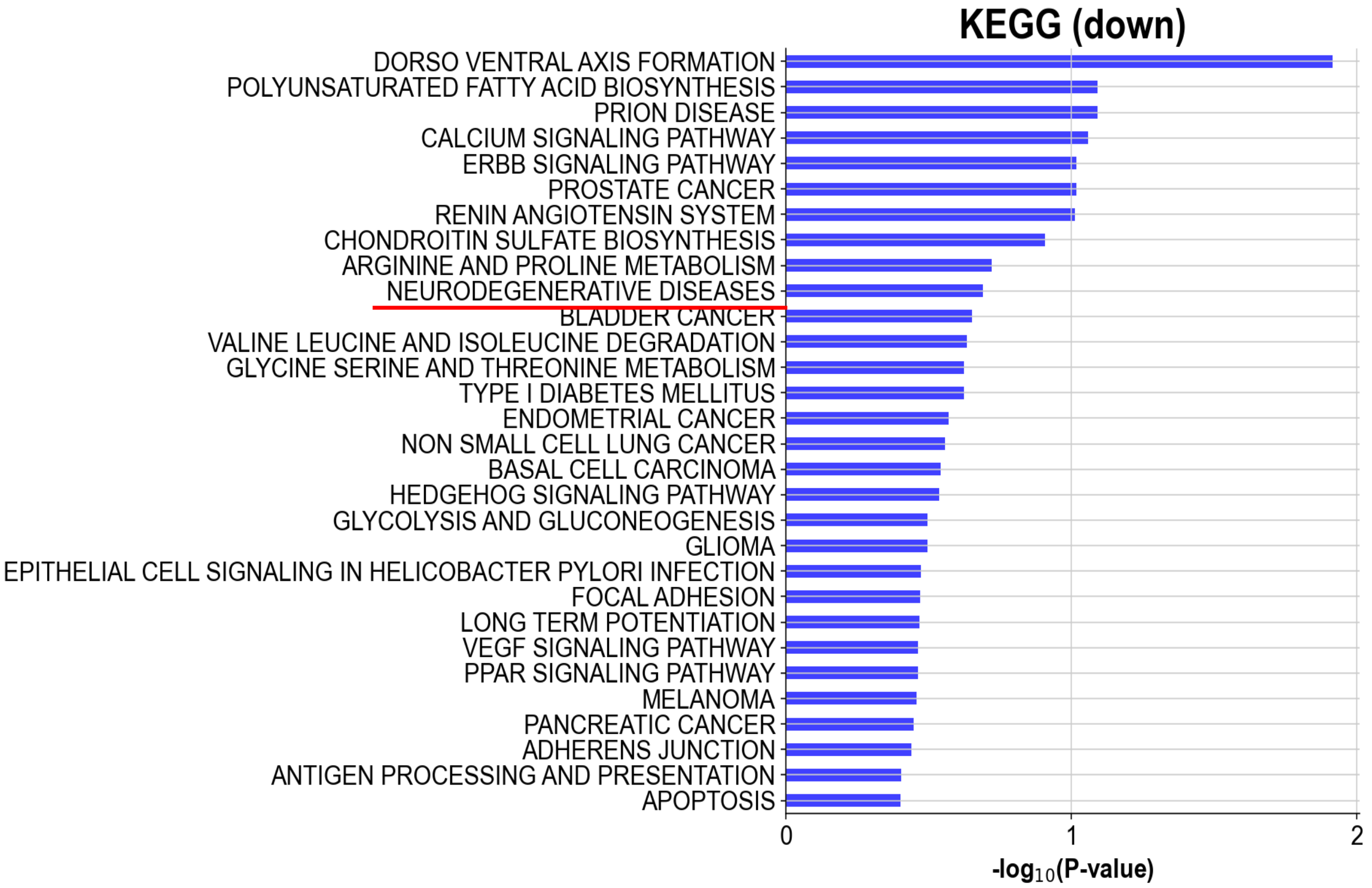

Supplementary Figure 17: GO terms enriched in the analysis of the downregulated DEGs of the glial population in patient cortical organoids with metformin treatment versus untreated organoids.

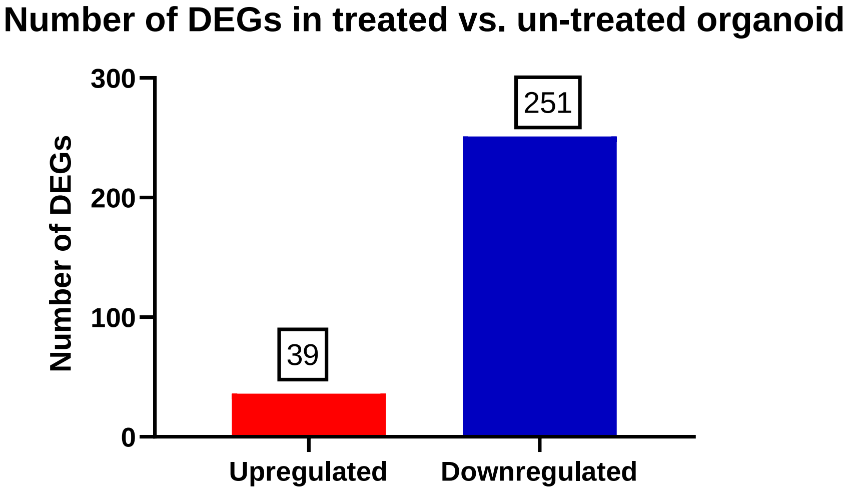

Supplementary Figure 17: Numbers of up- and downregulated DEGs pooled for the dopaminergic neuron population in metformin treated patient cortical organoids versus untreated organoids.

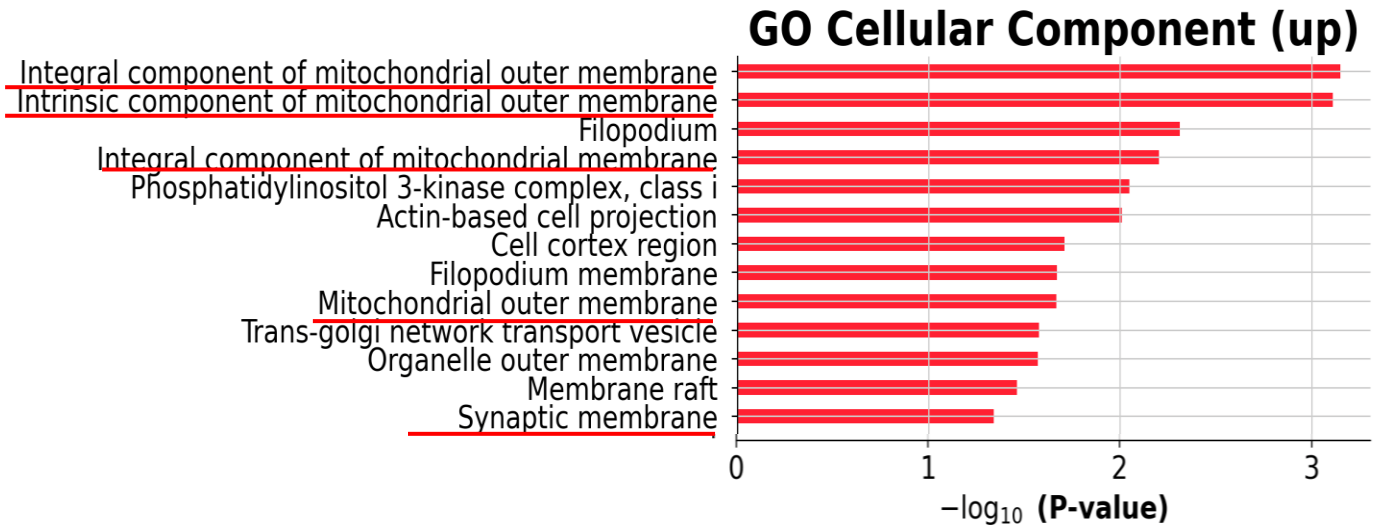

Supplementary Figure 18: GO cellular components enriched in the analysis of the upregulated DEGs of the dopaminergic neuron population in patient cortical organoids with metformin treatment versus untreated organoids.

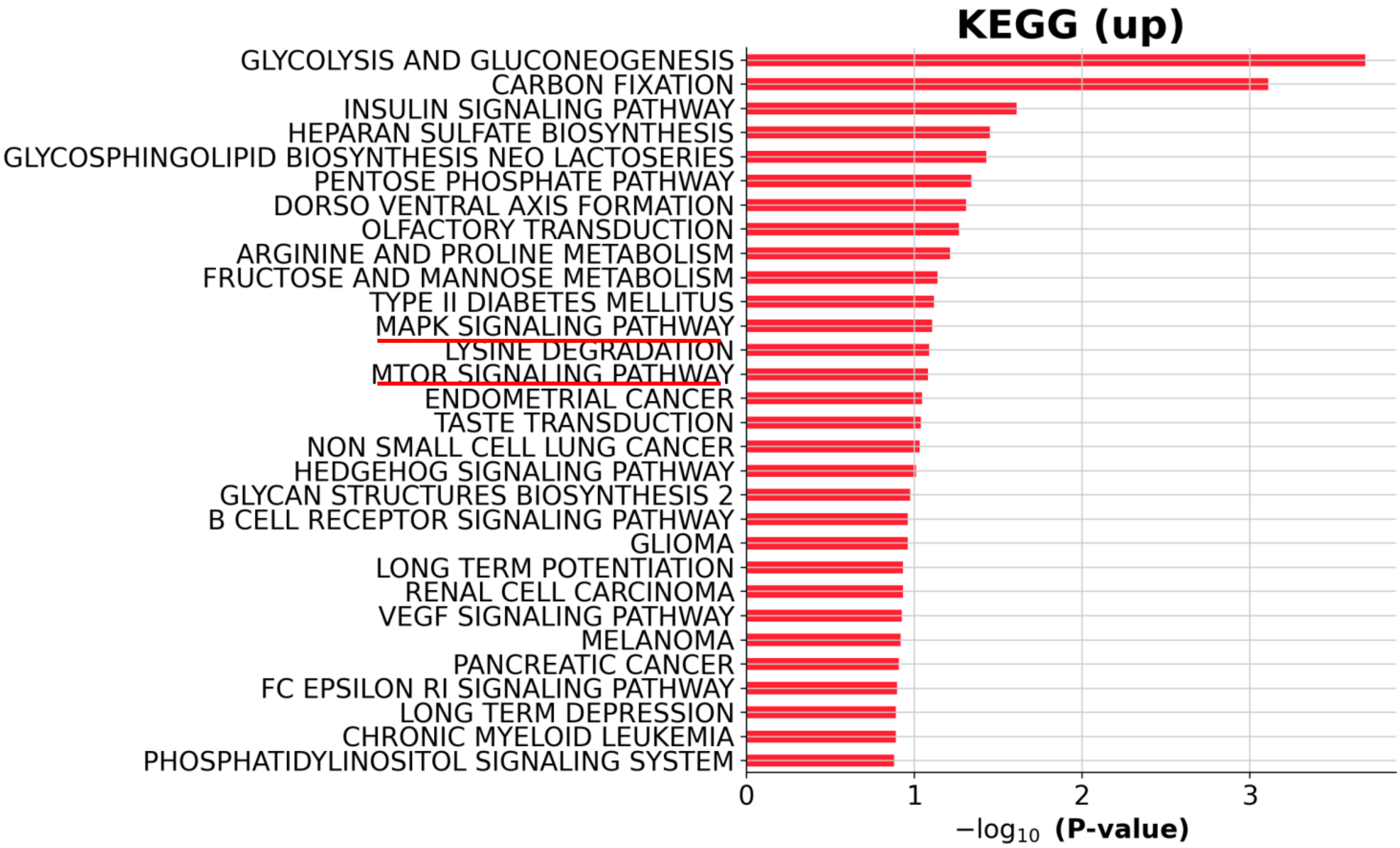

Supplementary Figure 19: KEGG pathways enriched in the analysis of the upregulated DEGs of the dopaminergic neuron population in patient cortical organoids with metformin treatment versus untreated organoids.

Supplementary Table 1: List of the information for the neural induction medium.

Component	Concentration	Supplier	Catalog number
**DMEM/F-12**		Life Technologies	11330057
**Knockout serum replacement**		Life Technologies	10828028
**MEM-NEAA**	1% (v/v)	Life Technologies	35050
**β-Mercaptoethanol**	100 μM	Sigma-Aldrich	M7522
**LDN-193189**	100 nM	Sigma-Aldrich	SML0559
**SB431542**	10 μM	Abcam	ab120163
**XAV939**	2 μM	Sigma-Aldrich	X3004

Supplementary Table 2: List of the information for the neural differentiation medium minus vitamin A.

Component	Concentration	Supplier	Catalog number
**DMEM/F-12**		Life Technologies	11330057
**Neurobasal medium**		Life Technologies	2110349
**Insulin**	0.025% (v/v)	Sigma-Aldrich	I9278
**MEM-NEAA**	0.5% (v/v)	Life Technologies	11140050
**Glutamax supplement**	1% (v/v)	Life Technologies	35050
**Penicillin/Streptomycin**	1% (v/v)	Life Technologies	15140-122
**N2 supplement**	0.5% (v/v)	Life Technologies	17502-048
**B27 supplement without vitamin A**	1% (v/v)	Life Technologies	12587010
**β-Mercaptoethanol**	50 µM	Sigma-Aldrich	M7522

Supplementary Table 3: List of the information for the neural differentiation medium minus vitamin A.

Component	Concentration	Supplier	Catalog number
**DMEM/F-12**		Life Technologies	11330057
**Neurobasal medium**		Life Technologies	2110349
**Insulin**	0.025% (v/v)	Sigma-Aldrich	I9278
**MEM-NEAA**	0.5% (v/v)	Life Technologies	11140050
**Glutamax supplement**	1% (v/v)	Life Technologies	35050
**Penicillin/Streptomycin**	1% (v/v)	Life Technologies	15140-122
**N2 supplement**	0.5% (v/v)	Life Technologies	17502-048
**B27 supplement**	1% (v/v)	Life Technologies	17504-04
**β-Mercaptoethanol**	50 μM	Sigma-Aldrich	M7522
**BDNF**	20 ng/ml	Pepro Tech	450-02
**Ascorbic acid**	200 μM	Sigma-Aldrich	A92902

Supplementary Table 4: List of the information on the software used for scRNA-seq analysis.

Analysis	Software	Version
**Pre-processing tool**	CeleScope	1.7.1
**Quality Control of reads**	fastqc	0.11.8
**Trimming**	cutadapt	1.17
**Alignment**	STAR	2.6.1a
**Quantification**	featureCounts	2.0.1
**Downstream analysis**	R	4.1.3
	python	3.9.12
	Seurat	3.1.2
	scanpy	1.9.1
	anndata	0.8.0
	leidenalg	0.8.10
	scvelo	0.2.2
	Monocle2	2.4.0
**Enrichment analysis**	gseapy	0.10.8
**Gene annotation**	scMRMA	1.0

Supplementary Table 5: The list of statistical tests and p-values in the figures.

Figure	Test	p-value
Fig. 2D	One-way ANOVA	** 0.0094
Fig. 2E	One-way ANOVA	ns 0.0848
Fig. 4D	Unpaired t test	*** 0.0006
Fig. 4E	Unpaired t test	*** 0.0001
Fig. 4F	Mann-Whitney test	* 0.0414
Fig. 4H	Unpaired t test	ns 0.1795
Fig. 4I	Unpaired t test	*** 0.0010
Fig. 4J	Mann-Whitney test	** 0.0016
Fig. 5B	Unpaired t test	****< 0.0001
Fig. 5C	Unpaired t test	ns 0.3425
Fig. 5D	Unpaired t test	****< 0.0001
Fig. 5F	Mann-Whitney test	****< 0.0001
Fig. 6J	Mann-Whitney test	*< 0.0286
Fig. 8C	Mann-Whitney test	ns 0.9451
Fig. 8D	Mann-Whitney test	**< 0.0015
Fig. 8E	Mann-Whitney test	**< 0.0061
Fig. 8G	Unpaired t test	***< 0.001
Fig. 8H	Unpaired t test	*< 0.0205
Fig. 9B	Mann-Whitney test	** 0.0080
Fig. 9C	Mann-Whitney test	***< 0.001
Fig. 9D	Unpaired t test	*** 0.0003
Fig. 9F	Unpaired t test	*** 0.0009
Fig. 9G	Mann-Whitney test	****< 0.0001

Supplementary Table 6: List of the information on the samples analyzed by scRNA-seq.

Sample	Estimated Number of Cells	Mean Reads per Cell	Median UMI per Cell	Total Gene	Median Gene per Cell
**Control organoid**	5,369	49,258	1,882	24,576	1,010
**patient organoid**	3,331	69,973	3,542	25,389	1,648
**patient organoid + metformin**	8,433	27,269	1,919	26,403	1,071

Supplementary Table 7: The list of top 10 genes for Figure 6A-D in cortical organoids of cell type enrichment analysis in scRNA-seq analysis.

DA GLU neurons	Dopaminergic neurons	Ependymal cells	GABAergic neurons	Glutaminergic neurons	Neural progenitor cells	Radial glia cells
SCG2	*NEUROD6*	*CA4*	*RAB8A*	*MT-CYB*	*RPS27*	*PTN*
NSG2	*TMSB10*	*WLS*	*PIGK*	*MT-ND5*	*FTL*	*C1orf61*
MMP3	*TUBA1A*	*MGST1*	*ZDHHC5*	*NEFM*	*RPL21*	*VIM*
ATP1B1	*SOX4*	*PRTG*	*FZD3*	*MT-ATP6*	*FTH1*	*CLU*
RTN4	*STMN2*	*PLS3*	*CCM2*	*MT-ND4*	*RPL15*	*HES1*
NEGR1	*PTMA*	*RSPO2*	*MRPL47*	*NEFL*	*RPL13A*	*HSPB1*
HSP90AA1	*NREP*	*FAM122B*	*PNRC1*	*MTND3*	*RPS5*	*MDK*
CELF4	*SOX11*	*WNT2B*	*RHBDD2*	*MT-ND2*	*CDKN1A*	*DBI*
LMO3	*MLLT11*	*CDO1*	*CDC42SE1*	*MT-CO3*	*VIM*	*LINC01158*

Supplementary Table 8: The list of GO terms and p-values in Supplementary Figure 5.

GO terms	p-value
Axon development	0.0000
SRP-dependent cotranslational protein targeting to membrane	0.0000
Cotranslational protein targeting to membrane	0.0000
Protein targeting to ER	0.0000
Nuclear-transcribed mRNA catabolic process, nonsense-mediated decay	0.0000
Cytoplasmic translation	0.0001
Peptide biosynthesis process	0.0002
Cell morphogenesis involved in neuron differentiation	0.0002
Nuclear-transcribed mRNA catabolic process	0.0002
Cardiac ventricle formation	0.0002
Generation of neurons	0.0007
Regulation of microtubule polymerization or depolymerization	0.0008
Protein depolymerization	0.0008
Translation	0.0008
Sequestering of actin monomers	0.0009
rRNA metabolic process	0.0010
rRNA processing	0.0015
Cellular macromolecule biosynthetic process	0.0015
Regulation of microtubule cytoskeleton organization	0.0017
Cellular protein metabolic process	0.0021
Regulation of neuron differentiation	0.0021
Ribosome biogenesis	0.0022
Neural tube development	0.0026
Positive regulation of neuron differentiation	0.0026
ncRNA processing	0.0026
Gene expression	0.0026
Microtubule depolymerization	0.0032
Neuron migration	0.0039

Supplementary Table 9: The list of Reactome terms and p-values in Supplementary Figure 6.

Reactome terms	p-value
Metabolism of proteins	0.0000
3'-UTR-mediated translational regulation	0.0000
Influenza infection	0.0000
Gene Expression	0.0012
Synaptic Transmission	0.0185

Supplementary Table 10: The list of top 10 genes for Figure 6E-H in patient cortical organoids of cell type enrichment analysis in scRNA-seq analysis.

GABAergic neurons	Glutaminergic neurons	Dopaminergic neurons	Neural progenitor cells	Melanocytes	Radial glial cell	Tanycytes	Fibroblasts	Ependymal cells	Astrocytes
H3F3B	*TCF7L2*	*RTN1*	*RPS19*	*MLANA*	*C1orf61*	*SPARCL1*	*LUM*	*CLU*	*ATP1A2*
NREP	*GAP43*	*NREP*	*RPS27*	*PMEL*	*PTN*	*GPM6B*	*COL3A1*	*SPARCL1*	*NTRK2*
SOX4	*STMN2*	*STMN2*	*EEF1A1*	*TYRP1*	*GPM6B*	*CLU*	*DCN*	*IFITM3*	*SLC1A3*
NR2F2	*MAP1B*	*PTMA*	*RPL37*	*ANXA2*	*VIM*	*PTN*	*LGALS1*	*SPARC*	*SPARCL1*
MLLT11	*STMN1*	*NSG2*	*RPL7*	*GPNMB*	*CLU*	*NTRK2*	*COL5A1*	*B2M*	*GPM6B*
EIF4G2	*SCG2*	*TUBA1A*	*RPLP0*	*DCT*	*PTPRZ1*	*ATP1A2*	*RPS18*	*RPS27L*	*PTN*
RTN	*NEFL*	*NOVA1*	*RPL18A*	*SAT1*	*CNN3*	*PSAT1*	*RPL37*	*SERF2*	*ADGRG1*
RBFOX2	*STMN4*	*MLLT11*	*C1orf61*	*QPCT*	*TTYH1*	*SLC1A3*	*RPL31*	*PLTP*	*CLU*
GAD1	*PGM2L1*	*TMSB10*	*RPL13A*	*LGALS1*	*EDNRB*	*SPARC*	*RPL13*	*MGST1*	*TTYH1*

Supplementary Table 11: The list of GO cellular component terms and p-values in Supplementary Figure 8.

GO cellular component	p-value
Dendrite (GO:0030425)	0.0033
Anchored component of plasma membrane (GO:0005886)	0.0254
Rdna heterochromatin (GO:0000781)	0.0265
Mpp7-dlg1-lin7 complex (GO:0030054)	0.0265
Endoplasmic reticulum-golgi intermediate compartment membrane (GO:0033116)	0.0286
Autolysosome (GO:0045324)	0.0525
Cmg complex (GO:1990429)	0.0527
Lamellar body (GO:0042628)	0.0674
Nuclear chromosome (GO:0000228)	0.0731
Neuron projection (GO:0043005)	0.0781
Secondary lysosome (GO:0005764)	0.0838
Axon (GO:0030424)	0.0977

Supplementary Table 12: The list of GO biological process terms and p-values in Supplementary Figure 9.

GO biological process terms	p-value
Development of Primary Male Sexual Characteristics: GO:0046545	0.0001
Male Gonad Development: GO:0008584	0.0001
Gonad Development: GO:0008406	0.0002
Sensory Organ Development: GO:0007423	0.0002
Positive Regulation of Cellular Metabolic Process: GO:0031325	0.0002
Positive Regulation of Kinase Activity: GO:0033674	0.0004
Negative Regulation of Cell Differentiation: GO:0045596	0.0006
Myeloid Leukocyte Differentiation: GO:0002573	0.0006
Central Nervous System Development: GO:0007417	0.0006
Axon Guidance: GO:0007411	0.0008
Regulation of Positive Chemotaxis: GO:0050927	0.0008
Negative Regulation of Transcription, DNA-Templated: GO:0045892	0.0015
Regulation of Kinase Activity: GO:0043549	0.0016
Negative Regulation of Calcineurin-NFAT Signaling Cascade: GO:2001241	0.0018
Axonal Fasciculation: GO:0007413	0.0018
Negative Regulation of Calcineurin-Mediated Signaling: GO:1901226	0.0018
Positive Regulation of Reactive Oxygen Species Metabolic Process: GO:2000379	0.0023
Positive Regulation of Phosphorylation: GO:0042327	0.0023
Regulation of Cell Population Proliferation: GO:0042127	0.0024
Ovarian Follicle Development: GO:0001541	0.0025
Nervous System Development: GO:0007399	0.0026
Neurotrophin TRK Receptor Signaling Pathway: GO:0048011	0.0028
Macrophage Differentiation: GO:0030225	0.0029
Regulation of Cyclin-Dependent Protein Kinase Activity: GO:0000079	0.0030
Regulation of Reactive Oxygen Species Metabolic Process: GO:2000377	0.0036
Positive Regulation of Cell Junction Assembly: GO:1904896	0.0036
Renal System Development: GO:0072001	0.0036
Regulation of Steroid Biosynthetic Process: GO:0019219	0.0036
Positive Regulation of Positive Chemotaxis: GO:0050921	0.0036
Chordate Embryonic Development: GO:0043009	0.0038

Supplementary Table 13: The list of GO molecular function processes and p-values in Supplementary Figure 11.

GO molecular function processes	p-value
Sequence-specific DNA binding (GO:0043565)	0.003193
Sequence-specific single stranded DNA binding (GO:0098847)	0.00723
Double-stranded DNA binding (GO:0003690)	0.013839
Semaphorin receptor activity (GO:0017154)	0.015839
Single-stranded telomeric DNA binding (GO:0043047)	0.017266
Sequence-specific double-stranded DNA binding (GO:1990837)	0.018649
RNA polymerase II core promoter sequence-specific DNA binding (GO:0000979)	0.020116
RNA polymerase II cis-regulatory region sequence-specific DNA binding (GO:0000978)	0.023298
Cis-regulatory region sequence-specific DNA binding (GO:0000987)	0.023298
Actin binding (GO:0003779)	0.027039
R-SMAD binding (GO:0070412)	0.027205
NAD+ adp-ribosyltransferase activity (GO:0003950)	0.037047
Transcription regulatory region nucleic acid binding (GO:0001067)	0.037639
Metallocarboxypeptidase activity (GO:0004181)	0.041235
RNA polymerase II transcription regulatory region sequence-specific DNA binding (GO:0000977)	0.043676
Telomeric DNA binding (GO:0042162)	0.046793
Core promoter sequence-specific DNA binding (GO:0001046)	0.052319
Pentosyltransferase activity (GO:0016763)	0.053695
Carboxypeptidase activity (GO:0004180)	0.053695
CCR chemokine receptor binding (GO:0048020)	0.059183
Cyclin-dependent protein serine/threonine kinase regulator activity (GO:0016538)	0.061915
Chemokine activity (GO:0008009)	0.06464
Chemokine receptor binding (GO:0042379)	0.070066
Receptor ligand activity (GO:0048018)	0.072694
Nuclear receptor coactivator activity (GO:0030374)	0.074115
Metalloexopeptidase activity (GO:0008235)	0.076806
Dna-binding transcription activator activity, RNA polymerase ii-specific (GO:0001228)	0.083621
Hormone activity (GO:0005179)	0.107207
DNA binding (GO:0003677)	0.111491
Growth factor activity (GO:0008083)	0.118838

Supplementary Table 14: The list of GO cellular component and p-values in Supplementary Figure 12.

GO cellular component	p-value
Apical dendrite (GO:0097440)	0.014409
Microtubule (GO:0005874)	0.028466
Cyclin-dependent protein kinase holoenzyme complex (GO:0000307)	0.042628
Cul4-ring E3 ubiquitin ligase complex (GO:0080008)	0.048177
Serine/threonine protein kinase complex (GO:1902554)	0.052319
Spindle microtubule (GO:0005876)	0.084831
Plasma membrane raft (GO:0044853)	0.112394
Intermediate filament cytoskeleton (GO:0045111)	0.114977
Early endosome membrane (GO:0031901)	0.13159
Asymmetric synapse (GO:0032279)	0.176037
Postsynaptic density (GO:0014069)	0.182034
Nucleus (GO:0005634)	0.184057
Cullin-ring ubiquitin ligase complex (GO:0031461)	0.20444
Membrane raft (GO:0045121)	0.211391
Cytoskeleton (GO:0005856)	0.215757
Spindle (GO:0005819)	0.244172
Axon (GO:0030424)	0.257348
Polymeric cytoskeletal fiber (GO:0099513)	0.311926
Early endosome (GO:0005769)	0.321968
Lytic vacuole membrane (GO:0098852)	0.322965
Dendrite (GO:0030425)	0.325945
Intracellular membrane-bounded organelle (GO:0043231)	0.330228
Endosome membrane (GO:0010008)	0.378398
Lysosomal membrane (GO:0005765)	0.382966
Microtubule cytoskeleton (GO:0015630)	0.383876
Collagen-containing extracellular matrix (GO:0062023)	0.4269
Focal adhesion (GO:0005925)	0.432804
Cell-substrate junction (GO:0030055)	0.438649

Supplementary Table 15: The list of top 10 genes for Figure 10A-D in patient cortical organoids with metformin treatment of cell type enrichment analysis in scRNA-seq analysis.

Dopaminergic neurons	Ependymal cells	Fibroblasts	GABAergic neurons	Glutaminergic neurons	Melanocytes	Neural progenitor cells	Radial glial cells	Tanycytes	Astrocytes
MAB21L1	*CLU*	*COL3A1*	*SOX4*	*TCF7L2*	*TYR*	*RPS27L*	*PTN*	*SPARCL1*	*ATP1A2*
PTMA	*IFITM3*	*COL1A2*	*LHX1*	*NEFL*	*TYRP1*	*RPS27*	*C1orf61*	*ATP1A2*	*SPARCL1*
RTN1	*SPARC*	*COL1A1*	*NREP*	*STMN2*	*DCT*	*RPS19*	*VIM*	*PTN*	*SLC1A3*
TMSB10	*SPARCL1*	*LGALS1*	*ZNF385D*	*GAP43*	*MLANA*	*FTL*	*GPM6B*	*CLU*	*PI15*
MLLT11	*B2M*	*ISLR*	*H3F3B*	*MAP1B*	*PMEL*	*GNG5*	*GNG5*	*NTRK2*	*QKI*
NREP	*NPC2*	*COL6A3*	*TFAP2A*	*STMN1*	*LGALS3*	*RPLP1*	*DBI*	*MGST1*	*PTPRZ1*
STMN2	*SERF2*	*LUM*	*RTN1*	*LHX9*	*PTGDS*	*RPL7*	*SPARC*	*IFITM3*	*GPM6B*
H3F3B	*GSTP1*	*MFAP4*	*BLCAP*	*NEFM*	*SAT1*	*VIM*	*CNN3*	*PLTP*	*PTN*
VSNL1	*CRYAB*	*POSTN*	*ETFB*	*WLS*	*CTSB*	*RPS3A*	*TTYH1*	*VIM*	*NTRK2*

Supplementary Table 16: The list of GO terms and p-values in Supplementary Figure 16.

GO terms	p-value
Regulation of neurotransmitter transport: GO:1902476	0.41704493
Regulation of sensory perception: GO:0051960	0.41704494
Regulation of dendritic cell apoptotic process: GO:2000252	0.41704495
Inositol phosphate dephosphorylation: GO:0043648	0.41704496
Columnar/cuboidal epithelial cell differentiation: GO:0002064	0.41704497
DNA damage response, signal transduction resulting in transcription: GO:0006978	0.41704498
Phosphorylated carbohydrate dephosphorylation: not a specific GO term	0.41704499
Enteric nervous system development: GO:0048484	0.41704500
Diterpenoid metabolic process: GO:0016103	0.41704501
Regulation of pseudopodium assembly: GO:0031274	0.41704502
Regulation of sister chromatid cohesion: GO:0031135	0.41704503
Dendritic cell chemotaxis: GO:1990736	0.41704504
Fat-soluble vitamin catabolic process: GO:0042364	0.41704505
Negative regulation of leukocyte apoptotic process: GO:2000271	0.41704506
Inositol phosphate catabolic process: GO:0016312	0.41704507
Positive regulation of pseudopodium assembly: GO:0031275	0.41704508
Cochlea morphogenesis: GO:0090102	0.41704509
Depurination: GO:0006284	0.41704510
Phasic smooth muscle contraction: not a specific GO term	0.41704511
Regulation of protein localization: GO:0032880	0.41704512
Positive regulation of lipid localization: GO:1905953	0.41704513
Response to prostaglandin E: GO:0071404	0.41704514
DNA damage response, signal transduction by p53 class mediator resulting in transcription of p21 class mediator: GO:0006979	0.41704515
Mitotic DNA replication: GO:1902275	0.41704516
Positive regulation of sequestering of triglyceride: not a specific GO term	0.41704517
Regulation of mitotic centrosome separation: GO:0051298	0.41704518
Very-low-density lipoprotein particle remodeling: GO:0034376	0.41704519
Embryonic forelimb morphogenesis: GO:0035115	0.41704520
Regulation of reactive oxygen species metabolic process: GO:2000377	0.41704521
Renal system development: GO:0072001	0.41704522

Supplementary Table 17: The list of KEGG pathways and p-values in Supplementary Figure 17.

KEGG pathways	p-value
Dorso ventral axis formation	0.0120
Polyunsaturated fatty acid biosynthesis	0.0806
Prion disease	0.0813
Calcium signaling pathway	0.0878
ERBB signaling pathway	0.0952
Prostate cancer	0.0952
Renin angiotensin system	0.0970
Chondroitin sulfate biosynthesis	0.1237
Arginine and proline metabolism	0.1908
Neurodegenerative diseases	0.2050
Bladder cancer	0.2222
Valine leucine and isoleucine degradation	0.2331
Glycine serine and threonine metabolism	0.2387
Type I diabetes mellitus	0.2387
Endometrial cancer	0.2702
Non small cell lung cancer	0.2754
Basal cell carcinoma	0.2861
Hedgehog signaling pathway	0.2916
Glycolysis and gluconeogenesis	0.3207
Glioma	0.3207
Epithelial cell signaling in helicobacter pylori infection	0.3396
Focal adhesion	0.3412
Long term potentiation	0.3412
VEGF signaling pathway	0.3445
PPAR signaling pathway	0.3445
Melanoma	0.3511
Pancreatic cancer	0.3596
Adherens junction	0.3682
Antigen processing and presentation	0.3936
Apoptosis	0.3974

Supplementary Table 18: The list of GO cellular component and p-values in Supplementary Figure 19.

GO cellular component	P-value
Integral component of mitochondrial outer membrane (GO:0031307)	0.000711
Intrinsic component of mitochondrial outer membrane (GO:0031306)	0.000778
Filopodium (GO:0030175)	0.004888
Integral component of mitochondrial membrane (GO:0032592)	0.006285
Phosphatidylinositol 3-kinase complex, class I (GO:0097651)	0.008968
Actin-based cell projection (GO:0098858)	0.009783
Cell cortex region (GO:0099738)	0.019627
Filopodium membrane (GO:0031527)	0.021393
Mitochondrial outer membrane (GO:0005741)	0.021573
Trans-golgi network transport vesicle (GO:0030140)	0.026671
Organelle outer membrane (GO:0031968)	0.026938
Membrane raft (GO:0045121)	0.034704
Synaptic membrane (GO:0097060)	0.04579
